# “Parental” responses to human infants (and puppy dogs): Evidence that the perception of eyes is especially influential, but eye contact is not

**DOI:** 10.1371/journal.pone.0232059

**Published:** 2020-05-06

**Authors:** Brandon M. Woo, Mark Schaller

**Affiliations:** 1 Department of Psychology, Harvard University, Cambridge, Massachusetts, United States of America; 2 Department of Psychology, University of British Columbia, Vancouver, British Columbia, Canada; Leiden University, NETHERLANDS

## Abstract

The present investigation tests: (i) whether the perception of an human infant’s eyes, relative to other facial features, especially strongly elicits “parental” responses (e.g., appraisals of cuteness and vulnerability); (ii) if, so, whether effects of the visual perception of eyes may be partially attributable to eye contact; (iii) whether the perception of non-human animals’ (puppy dogs’) eyes also especially strongly influence appraisals of their cuteness and vulnerability; and (iv) whether individual differences in caregiving motives moderate effects. Results from 5 experiments (total *N* = 1458 parents and non-parents) provided empirical evidence to evaluate these hypotheses: Appraisals of human infants were influenced especially strongly by the visual perception of human infants’ eyes (compared to other facial features); these effects do *not* appear to be attributable to eye contact; the visual perception of eyes influenced appraisals of puppy dogs, but not exactly in the same way that it influenced appraisals of human infants; and there was no consistent evidence of moderation by individual differences in caregiving motives. These results make novel contributions to several psychological literatures, including literatures on the motivational psychology of parental care and on person perception.

## Introduction

Human development includes a long infancy phase during which offspring cannot fend for themselves. As a consequence, a set of psychological mechanisms—a parental care motivational system—evolved to facilitate caregiving responses to young children [1–3]. These mechanisms operate in non-parents as well as parents, and respond to the perception of features diagnostic of infancy [4,5]. Lorenz [6] proposed that these features elicit increased caregiving responses in adults. To the extent that people (and non-human animals) are characterized by such babyish features, they are judged to be cuter, younger, and more vulnerable, and elicit more prototypically parental behavioral responses [7–10].

Although there are many different facial features that may be diagnostic of infancy (e.g., large eyes, small nose, narrow jaw, round cheeks), some features may be especially important in eliciting “parental” responses from both parents and non-parents. In this article, we report: (i) three experiments testing the hypothesis that the visual perception of human infants’ eyes plays an especially important role in eliciting appraisals of cuteness and vulnerability; and (ii) two additional experiments that test the hypothesis that this effect is due specifically to the psychological consequences of eye contact. Additionally, across all five experiments, we test whether these effects emerge when perceiving juvenile non-human animals (puppy dogs), and whether effects are moderated by individual differences in activation of the parental care motivational system.

### Are subjective appraisals of human infants influenced especially strongly by their eyes?

Previous studies have tested how appraisals of children’s cuteness are influenced by the physical dimensions of their facial features, and found that faces with larger eyes, larger pupils, and smaller noses are judged to be cuter [11–13]. Although these results reveal that appraisals of cuteness are influenced by specific physical properties of human infants’ eyes, it remains unclear: (i) whether the mere perception of eyes—compared to the perception of other facial features (e.g., the nose)—plays an outsized role in these appraisals; and (ii) the extent to which the perception of eyes might have an especially strong influence on appraisals of other qualities (e.g., vulnerability) that motivate caregiving responses.

There are reasons that the perception of eyes might be uniquely influential in guiding these appraisals [14]. When looking at faces, perceivers allocate more attention to eyes than to other facial features [15–17], suggesting that perceivers may tacitly perceive eyes to be uniquely informative. If so, perceivers may accord special status to eyes when appraising the functionally relevant qualities of other people. This general thesis has received some support in other domains of person perception. For example, compared to other facial features, information contained within eyes has the greatest influence on appraisals of a face’s “humanness” [18–19]. These appraisals of humanness may be based, in part, on appraisals of underlying mental capabilities [19]. Given that attributions about mental capabilities may also be associated with subjective appraisals of cuteness [20], these results indirectly attest to the possibility that visual perception of human infants’ eyes may be especially influential in guiding appraisals of cuteness and vulnerability.

Previous research linking specific facial features to appraisals of cuteness [11,13] manipulated specific physical dimensions of specific features, such as the width of the eyes or the length of the nose. Such an approach tests the extent to which appraisals are influenced by those specific dimensions of those specific features, but does not directly test whether a particular facial feature itself (such as the eyes) might—for reasons including but not limited to its physical dimensions—have an especially potent influence on appraisals. Therefore, we used a different methodological approach, in which we manipulated whether specific facial features (such as the eyes) were—or were not—visible at all, and tested whether this manipulation influenced perceivers’ subjective appraisals of cuteness and vulnerability.

### Are subjective appraisals of human infants influenced by eye contact?

If the perception of human infants’ eyes plays an outsized role in guiding appraisals, this effect may reflect diagnostic information implied by the physical properties of eyes; but it is also possible that eyes might also be influential because of what they might be looking *at*—especially if those eyes are looking at the perceiver. Eye gaze can provide diagnostic information about individuals’ mental states [14], and eye contact has well-documented implications for social cognition and person perception [21–23]. Many functional appraisals are amplified if a target gazes directly at the perceiver rather than away. For instance, among adults in relationships, eye contact is associated with stronger romantic love [24]; and within mother-child relationships, eye contact is associated with stronger maternal oxytocin responses—indicative of stronger maternal bonds [25]. Given these findings it seems plausible that, if the perception of eyes exerts an especially powerful influence on appraisals of children’s cuteness and vulnerability, this effect might occur, in part, because eyes provide the opportunity for eye contact. If so, then (compared to human infants whose gaze is averted) human infants gazing directly at the perceiver may be judged to be cuter and more vulnerable.

### Do the perception of eyes and eye contact influence appraisals of non-human animals?

Babyish features may be diagnostic of infancy, but not perfectly so. Some adults are characterized by these features; and, because motivational mechanisms of parental care are responsive to superficial cues, these baby-faced adults elicit appraisals mimicking those of young children [26–27]. Similarly, babyish features can be characteristic of many juvenile non-human animals, with analogous implications for appraisals (e.g., people perceive kittens to be cuter than adult cats) [12]. Although not all responses to human infants and juvenile non-human animals are comparable (e.g., evidence suggests that visual attention may be preferentially captured by babyish features in human faces, but not by babyish features in the faces of cats and dogs) [28], some psychological phenomena associated with the perception of human infants do appear to be similar to the psychological phenomena associated with the perception of juvenile mammals (e.g., the perception of cuteness for human infants) [10,15,29]. Therefore, the experiments reported below tested whether the perception of eyes plays an outsized role in guiding appraisals of both human infants and juvenile non-human animals. To do so, we assessed the effects of eyes and eye gaze on appraisals of both human infants and puppy dogs.

### Are the effects moderated by individual differences in parental care motives?

Appraisals of children’s cuteness and vulnerability may reflect motivational mechanisms associated with parental caregiving. Although this parental care motivational system appears to be part of the cognitive architecture of all humans (including non-parents), there are individual differences in the extent to which people experience the protective and nurturant tendencies that characterize it [26,30]. These individual differences influence responses to children and to child-like beings (e.g., baby-faced adults), and can moderate the effects of other variables on these responses. For instance, although people generally find it more subjectively rewarding to look at the faces of cuter (vs. less cute) babies, this effect is moderated by self-reported parental tendencies [31].

In our experiments, we assessed individual differences in the parental care motivational system and tested whether these individual differences moderated effects of eye perception and eye contact on appraisals of human infants and puppy dogs. To do so, we employed a measure that assesses two conceptually distinct kinds of differences in parental care motives—inclinations to protect and inclinations to nurture for [30].

### Overview of experiments

Five experiments addressed the research questions identified above. Experiments 1–3 tested whether the perception of eyes—compared to the perception of other facial features—is especially influential in guiding “parental” appraisals of young children and puppy dogs. Participants (including both parents and non-parents) appraised images of either human infants or puppy dogs; the images were manipulated such that either eyes were visible but other babyish features (nose, mouth) were not, or these other facial features were visible but eyes were not. Across these experiments, two different kinds of stimuli were employed (photographs and cartoons) and multiple methods were used to manipulate the visibility of eyes versus other babyish features.

Experiments 4 and 5 focused on effects of eye contact. Participants appraised images of either human infants or puppy dogs; the images were retouched such that the target object (human infant or puppy dog) either looked at or away from perceivers. Across these two experiments, two different kinds of stimuli were employed (photographs and cartoons).

In all experiments, participants completed a self-report questionnaire [30] assessing individual differences in the nurturant and protective tendencies associated with the parental care motivational system.

## Experiment 1

### Methods

All data are available at osf.io/592xe/?view_only=4c0c3cb804db44a59d6d004af6bd53e3.

#### Participants

Participants were 306 adults in the United States (*M* age = 34.94 years; 141 women; 103 parents; 134 dog-owners) who were recruited online on Amazon’s Mechanical Turk (www.mturk.com). Participants were randomly assigned to view photographs depicting either human infants (*n* = 153) or puppy dogs (*n* = 153). Exploratory analyses revealed that a sample of 130 would have sufficient power to detect a medium-sized effect (within-subjects differences in responses) with > .99 power and α = .05. We assumed a medium-sized effect given past work suggestive that physical similarity to human infants influences cuteness perception at a medium effect size [12]. Thus, we recruited at least 130 participants for all experiments in this paper.

For all experiments, participants gave consent to complete the tasks online. The study and consent procedures for all experiments were approved by the University of British Columbia Behavioural Research Ethics Board.

#### Appraisals of human infants/puppy dogs

**Fig 1:**
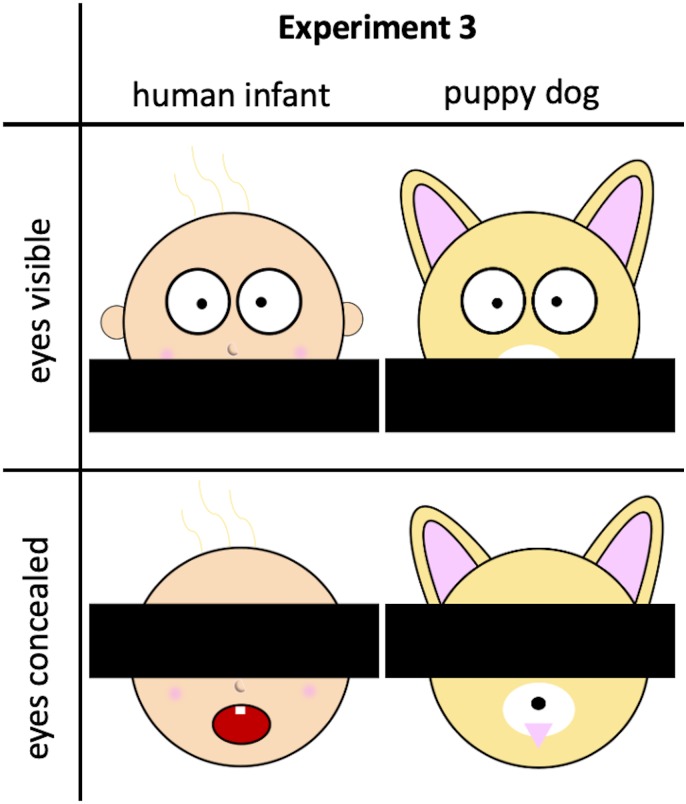
Example stimuli from Experiment 3. We are unable to share examples of the stimuli from Experiments 1 and 2 because of copyright issues.

Participants were presented serially with eight photographs depicting either human infants or puppy dogs. Superimposed on each image was a black rectangle. For four images, the rectangle concealed either the nose or the mouth of the baby/puppy dog, while leaving the rest of the face (including the eyes) visible. For the other four images, the rectangle concealed the eyes, while leaving the rest of the face visible. Fig 1 depicts examples of stimuli that are similar to the stimuli that participants saw. The original photographs were obtained via Google searches with the terms “cute baby” and “cute puppy.” (Human infants in the photographs were neutral in expression and approximately 6–12 months in age. Photographs were cropped so that the face was central; the upper body may have been visible for some stimuli, but was equally visible regardless of whether the eyes were concealed.) Other than the manipulation that was imposed upon these images, all participants in the human infants condition saw the same images and all participants in the puppy dogs condition saw the same images. Across participants, we counterbalanced the images for which eyes were visible/concealed, and also counterbalanced order of presentation. Participants rated each target according to: “how cute” it was, “how vulnerable” it was, “how self-reliant” it was, and how much participants felt “a need to protect” it. Ratings were made on 10-point scales (1 = *not at all*; 10 = *extremely*).

#### Individual differences in parental care and tenderness

In addition to completing a questionnaire assessing demographic information (e.g., sex, parental status, dog ownership, etc.), participants also completed a 10-item version of the Parental Care and Tenderness questionnaire (PCAT) [30]. Six items assessed nurturant responses toward young children (e.g., “Babies melt my heart”) and 4 items assessed protective responses toward young children (e.g., “I would use any means necessary to protect a child, even if I had to hurt others”). We computed subscale scores for *PCAT-Nurturance* and *PCAT-Protection* (Cronbach’s α = .90 and .89, respectively).

#### Statistical analysis

For all analyses in our experiments, we used R version 3.6.1. For all analyses, the threshold of significance was set at α = .05.

### Results

#### Effects of eye visibility manipulation on appraisals of human infants and puppy dogs

Table 1 summarizes mean ratings of human infants’ and puppy dogs’ cuteness, vulnerability, self-reliance, and need for protection, depending on whether eyes were visible or concealed. We conducted mixed-effects models on these ratings. Fixed effects were the eye visibility manipulation (-0.5 = eyes concealed, 0.5 = eyes visible), target type (-0.5 = human infant, 0.5 = puppy dog), and the interaction between these two variables. Random effects were participants and stimulus images. For all the output of the models on the effects of eye visibility in Study 1, see S1 Table.

**Table 1:** Experiment 1: Mean ratings (with standard deviations in parentheses) of human infants and puppy dogs, as a function of whether their eyes were visible or concealed.

	Ratings of Human Infants		Ratings of Puppy Dogs	
	Eyes Visible	Eyes Concealed	Eyes Visible	Eyes Concealed
	(Nose/Mouth Concealed)	(Nose/Mouth Visible)	(Nose/Mouth Concealed)	(Nose/Mouth Visible)
Cuteness	8.12	7.65	8.78	8.76
	(2.33)	(2.44)	(1.53)	(1.57)
Vulnerability	9.21	9.11	7.89	7.76
	(1.51)	(1.74)	(1.91)	(1.98)
Self-reliance	1.99	1.92	4.24	4.28
	(2.20)	(2.11)	(2.19)	(2.21)
Protection	8.17	8.08	7.72	7.69
	(2.47)	(2.51)	(2.22)	(2.24)

First, we examined ratings of cuteness. Targets were rated as cuter if their eyes were visible (β = 0.05, *t*(2127) = 7.13), *p* < .001, 95% CI [0.04, 0.07]), and puppy dogs were rated as cuter than human infants (β = 0.21, *t*(254) = 3.96, *p* < .001, 95% CI [0.10, 0.32]). These main effects were qualified by an interaction between eye visibility and target type (β = -0.05, *t*(2127) = -6.69, *p* < .001, 95% CI [-0.07, -0.03]). Post-hoc pairwise tests, correcting for multiple comparisons using Holm’s method, revealed that human infants were rated as cuter if their eyes were visible (*t*(2129) = -9.77, *p* < .001); in contrast, the eye visibility manipulation had no statistically significant effect on ratings of puppy dogs’ cuteness (*t*(2129) = -0.30, *p* = .757).

Second, we examined ratings of vulnerability. Targets were rated as more vulnerable if their eyes were visible (β = 0.03, *t*(2127) = 3.55, *p* < .001, 95% CI [0.01, 0.04]), and human infants were rated as more vulnerable than puppy dogs (β = -0.34, *t*(297) = -7.09, *p* < .001, 95% CI [-0.44, -0.25]). There was no statistically significant interaction (β = 0.00, *t*(2127) = 0.42, *p* = .644, 95% CI [-0.01, 0.02]).

Third, we examined ratings of self-reliance. Puppy dogs were rated as more self-reliant than human infants (β = 0.46, *t*(280) = 9.57, *p* < .001, 95% CI [0.37, 0.58]). There was no statistically significant main effect of the eye visibility manipulation (β = 0.00, *t*(2127) = 0.35, *p* = .725, 95% CI [-0.01, 0.01]), nor was there a statistically significant interaction (β = -0.01, *t*(2127) = -1.42, *p* = .153, 95% CI [-0.02, 0.003]).

Lastly, we examined ratings of how much participants felt a need to protect a target. Neither main effect was statistically significant (eye visibility: β = 0.01, *t*(2127) = 1.86, *p* = .062, 95% CI [-0.0005, 0.02]; target type: β = -0.08, *t*(311) = -1.58, *p* = .114, 95% CI [-0.19, 0.02]), nor was there a statistically significant interaction (β = -0.0007, *t*(2127) = -1.25, *p* = .210, 95% CI [-0.01, 0.0004]).

#### Analyses testing for moderating effects of PCAT

To test whether any of the preceding effects were moderated by PCAT, we conducted additional mixed-effects models that, in addition to the fixed effects and random effects identified above, also included the following fixed effects: The two PCAT subscales (PCAT-Nurturance, PCAT-Protection), as well as the two-way and three-way interactions between each subscale and the target type and eye visibility manipulations. Results revealed that only on ratings of cuteness was there any evidence of a statistically significant interaction between either PCAT subscale and the eye visibility manipulation (for all the output from the models, see S2, S3, S4 and S5 Tables). Specifically, there was a 2-way interaction between PCAT-Nurturance and the eye visibility manipulation (β = -0.09, *t*(2127) = -2.31, *p* = .020, 95% CI [-0.17, -0.01]), indicating that the effect of eye visibility on perceived cuteness was stronger among individuals who scored lower on PCAT-nurturance.

#### Exploratory analyses testing for moderating effects of demographic variables

Additionally, given that demographic variables (e.g., sex, parental status) may affect responses to infants and to babyish features [9,26] we also conducted analyses to test whether any of the primary findings were moderated by sex, parental status, and dog ownership. These analyses revealed three statistically significant 2-way interactions involving the eye visibility manipulation. For ratings of cuteness, there was an interaction between the eye visibility manipulation and parental status (*p* = .035): both parents and non-parents rated targets as cuter if their eyes were visible vs. concealed (*p*s = .011 and < .001, respectively), but the manipulation influenced non-parents’ ratings more strongly. Also on ratings of cuteness, there was an interaction between the eye visibility manipulation and dog ownership (*p* = .023): Both dog-owners and non-dog-owners rated targets as cuter if their eyes were visible vs. concealed (both *p*s < .001), but the manipulation influenced dog-owners’ ratings more strongly. For ratings of the need to protect the target, there was an interaction between the eye visibility manipulation and gender (*p* = .005): Women rated targets as more in need of protection if their eyes were visible vs. concealed (*p* = .001), but the effect of manipulation on men’s ratings was negligible (*p* = .473). Full details are presented in supplementary material (see S1 Text).

### Summary of primary results from Experiment 1

These results provide some evidence indicating that appraisals of human infants may be especially strongly influenced by perception of human infants’ eyes, compared to other prototypic babyish features (nose, mouth). Compared to conditions in which their eyes were concealed (but other facial features were visible), when their eyes were visible (but other facial features were concealed), human infants were judged to be cuter and more vulnerable.

The effect on appraisals of cuteness was specific to ratings of human infants; there was no such effect with ratings of puppy dogs. In contrast, the (weaker) effect on appraisals of vulnerability was observed for ratings of both human infants and puppy dogs. There was some evidence that the effects on appraisals of cuteness may be moderated by individual differences in parental nurturance.

## Experiment 2

Experiment 2 provided a conceptual replication of Experiment 1. The experimental design was identical, but Experiment 2 employed a different method to manipulate eye visibility: cropping images.

### Methods

#### Participants

Participants were 301 adults residing in the United States (*M* age = 37.93 years; 119 women, 1 other sex; 123 parents, 136 dog-owners) who were recruited on Mechanical Turk. Participants were randomly assigned to view photographs depicting either human infants (*n* = 149) or puppy dogs (*n* = 152).

#### Appraisals of human infants/puppy dogs

Participants were presented serially with eight images depicting either human infants or puppy dogs. Four images were cropped such that only the upper half of the face was visible, including the eyes. The other four images were cropped such that only the lower half of the face was visible (i.e., the nose, mouth, cheeks, and chin), thus excluding the eyes. The uncropped images were identical to the original photographs of Experiment 1. Fig 1 depicts examples of stimuli that are similar to the stimuli that participants saw. Other than the manipulation that was imposed upon these images, all participants in the human infants condition saw the same images and all participants in the puppy dogs condition saw the same images. Across participants, we counterbalanced the images for which eyes were visible/concealed, and also counterbalanced order of presentation. Participants’ appraisals of human infants/puppy dogs were assessed with the same rating measures employed in Experiment 1.

#### Individual differences in parental care and tenderness

Participants completed the same individual difference measures used in Experiment 1, including the PCAT measure that allowed us to compute scores on two subscales—PCAT-Nurturance and PCAT-Protection (both Cronbach’s αs = .91).

### Results

#### Effects of eye visibility manipulation on appraisals of human infants and puppy dogs

Table 2 summarizes mean ratings of human infants and puppy dogs, depending on whether the photographs included eyes. We conducted mixed-effects models identical to those of Experiment 1 (for all output, see S6 Table).

**Table 2:** Experiment 2: Mean ratings (with standard deviations in parentheses) of human infants and puppy dogs, as a function of whether their eyes were visible or not.

	Ratings of Human Infants		Ratings of Puppy Dogs	
	Eyes Visible	Eyes Not Visible	Eyes Visible	Eyes Not Visible
	(Lower Face Cropped Out)	(Upper Face Cropped Out)	(Lower Face Cropped Out)	(Upper Face Cropped Out)
Cuteness	8.09	7.90	8.14	7.89
	(1.99)	(2.00)	(1.78)	(1.73)
Vulnerability	9.25	9.20	7.13	6.63
	(1.41)	(1.45)	(2.08)	(2.12)
Self-reliance	1.80	1.85	5.06	5.39
	(1.82)	(1.88)	(2.29)	(2.25)
Protection	8.06	8.04	7.04	6.81
	(2.61)	(2.57)	(2.58)	(2.60)

First, we examined ratings of cuteness. Targets were rated as cuter if their eyes were visible (β = 0.05, *t*(2092) = 5.31, *p* < .001, 95% CI [0.03, 0.07]). There was no statistically significant main effect of target type (β = 0.00, *t*(167) = 0.09, *p* = .924, 95% CI [-0.10, 0.11]); nor was there a statistically significant interaction (β = 0.00, *t*(2092) = 0.75, *p* = .454, 95% CI [-0.01, 0.02]).

Second, we examined ratings of vulnerability. Targets were rated as more vulnerable if their eyes were visible (β = 0.06, *t*(2092) = 6.68, *p* < .001, 95% CI [0.04, 0.08]), and human infants were rated as more vulnerable than puppy dogs (β = -0.54, *t*(140) = -11.65, *p* < .001, 95% CI [-0.63, -0.45]). In addition, there was a statistically significant interaction (β = 0.05, *t*(2092) = 5.49, *p* < .001, 95% CI [0.03, 0.07). Post-hoc pairwise tests, corrected using Holm’s method, revealed that puppy dogs were rated as more vulnerable if their eyes were visible (*t*(2094) = -8.65, *p* < .001), but the eye visibility manipulation had no statistically significant effect on ratings of human infants’ vulnerability (*t*(2095) = -0.84, *p* = .401).

Third, we examined ratings of self-reliance. Targets were rated as less self-reliant if their eyes were visible (β = -0.03, *t*(2092) = -4.80, *p* < .001, 95% CI [-0.05, -0.02]), and puppy dogs were rated as more self-reliant than human infants (β = 0.63, *t*(237) = 14.92, *p* < .001, 95% CI [0.55, 0.71]). There was also a statistically significant interaction (β = -0.02, *t*(2092) = -3.51, *p* < .001, 95% CI [-0.04, -0.01]). Post-hoc pairwise tests, corrected using Holm’s method, revealed that puppy dogs were rated as less self-reliant if their eyes were visible (*t*(2094.5) = 5.90, *p* < .001), but the eye visibility manipulation had no statistically significant effect on ratings of human infants’ self-reliance (*t*(2095.8) = 0.90, *p* = .363).

Lastly, we examined ratings of how much participants felt a need to protect a target. Participants reported a stronger need to protect targets if their eyes were visible (β = 0.02, *t*(2092) = 4.55, *p* < .001, 95% CI [0.01, 0.04]); participants also reported a stronger need to protect human infants compared to puppy dogs (β = -0.20, *t*(306) = -3.76, *p* < .001, 95% CI [-0.31, -0.09]). Additionally, there was a significant interaction (β = 0.02, *t*(2092) = 3.86, *p* < .001, 95% CI [0.01, 0.03]). Post-hoc pairwise tests, corrected using Holm’s method, revealed that participants reported a stronger need to protect puppy dogs if their eyes were visible (*t*(2092.7) = -5.98, *p* < .001), but the eye visibility manipulation had no statistically significant effect on reported need to protect human infants (*t*(2093.2) = -0.48, *p* = .630).

#### Analyses testing for moderating effects of PCAT

To test whether any effects of the eye visibility manipulation were moderated by PCAT, we used the same analytic strategy employed in Experiment 1 (see S6, S7, S8, S9 and S10 Tables). Results revealed two notable statistically significant interaction effects: There was a 2-way interaction between PCAT-Protection and the eye visibility manipulation on ratings of cuteness (β = 0.22, *t*(2092.5) = 4.22, *p* < .001, 95% CI [0.12, 0.33]), and also on ratings of need to protect (β = 0.08, *t*(2093) = 2.47, *p* = .013, 95% CI [0.01, 0.14]). These interactions show that the effects of eye visibility on the perceived cuteness of targets and on the need to protect targets occurred more strongly among individuals who scored higher on the PCAT-Protection subscale.

#### Exploratory analyses testing for moderating effects of demographic variables

Analyses testing for moderating effects of demographic variables revealed only one statistically significant interaction involving the eye visibility manipulation. For ratings of cuteness, there was a 3-way interaction between the eye visibility manipulation, target type, and gender: Women rated puppy dogs as cuter when their eyes were visible vs. concealed (*p* < .001); in contrast, the manipulation did not influence women’s ratings of human infants (*p* = .464), or men’s ratings of either human infants (*p* = .151) or puppy dogs (*p* = .422). Full details are presented in supplementary material (see S1 Text).

### Summary of primary results from Experiment 2

Complementing Experiment 1, Experiment 2 used a different method to manipulate the visibility of eyes versus other babyish features. The results conceptually replicated one of the primary findings from Experiment 1: Human infants were rated as cuter under conditions in which their eyes were visible (and other babyish features were not), compared to conditions in which other babyish features were visible (and eyes were not). In contrast to Experiment 1, the results of Experiment 2 revealed this effect also on cuteness ratings of puppy dogs. Additionally, when puppy dogs’ (but not human infants’) eyes were visible, they were appraised as less self-reliant and more vulnerable, and participants reported a stronger need to protect them. As in Experiment 1, there was some evidence to suggest that the effects of eye visibility might be moderated by individual differences in parental care motives, but the specific moderating effects observed in Experiment 2 differed from the one moderating effect observed in Experiment 1.

## Experiment 3

Experiment 3 provided another conceptual replication of Experiments 1 and 2. The experimental design was identical, but rather than employing photographic stimuli (as in Experiments 1 and 2), participants in Experiment 3 were presented with cartoon drawings of human infants’ and puppy dogs’ faces. The specific method of manipulating eye visibility was the same as in Experiment 1.

### Methods

#### Participants

Participants were 280 adults residing in the United States (*M* age = 36.12 years; 155 women; 110 parents; 123 dog-owners) who were recruited on Mechanical Turk. Participants were randomly assigned to view cartoon images depicting either human infants (*n* = 141) or puppy dogs (*n* = 139).

#### Appraisals of human infants/puppy dogs

Participants were presented serially with four cartoon images. (These images were created for this study.) Superimposed on each image was a black rectangle. For 2 images, the rectangle concealed the mouth of the human infant/puppy dog, while leaving the rest of the face (including the eyes) visible; for the other 2 images, the rectangle concealed the eyes, while leaving the rest of the face visible. Fig 1 depicts examples of stimuli. Other than the manipulation that was imposed upon these images, all participants in the human infants condition saw the same images and all participants in the puppy dogs condition saw the same images. Across participants, we counterbalanced the images for which eyes were visible/concealed, and also counterbalanced order of presentation. Participants rated each human infant/puppy dog on 3 of the 4 rating measures employed in Experiments 1 and 2, assessing appraisals of “how cute” it was, “how vulnerable” it was, and how much they felt “a need to protect” it. (In contrast to Experiments 1 and 2, ratings of human infants’/puppy dogs’ self-reliance was not assessed in Experiments 3, 4 or 5).

#### Individual differences in parental care and tenderness

Participants completed the same individual difference measures used in Experiments 1 and 2. We computed participants’ scores on PCAT-Nurturance and PCAT-Protection (Cronbach’s αs = .91 and .88, respectively).

### Results

#### Effects of eye visibility manipulation on appraisals of human infants/puppy dogs

Table 3 summarizes mean ratings of human infants and puppy dogs, depending on eye visibility. We conducted mixed-effects models identical to those of Experiments 1 and 2 (for all output, see S11 Table).

**Table 3:** Experiment 3: Mean ratings (with standard deviations in parentheses) of human infants and puppy dogs, as a function of whether their eyes were visible or concealed.

	Ratings of Human Infants		Ratings of Puppy Dogs	
	Eyes Visible (Lower Face Concealed)	Eyes Concealed (Lower Face Visible)	Eyes Visible (Lower Face Concealed)	Eyes Concealed (Lower Face Visible)
Cuteness	6.07	5.06	5.88	6.16
	(2.38)	(2.58)	(2.15)	(2.05)
Vulnerability	6.82	6.66	5.67	5.38
	(2.56)	(2.77)	(2.40)	(2.37)
Protection	5.88	5.66	5.14	5.05
	(3.00)	(3.09)	(2.70)	(2.66)

First, we examined ratings of cuteness. Targets were rated as cuter if eyes were visible (β = 0.07, *t*(833) = 4.68, *p* < .001, 95% CI [0.04, 0.11]). There was no statistically significant difference in ratings of puppy dogs versus human infants (β = 0.09, *t*(90) = 1.78, *p* = .077, 95% CI [-0.009, 0.20]), but there was a statistically significant interaction (β = -0.13, *t*(833) = -8.18, *p* < .001, 95% CI [-0.17, -0.10]). Post-hoc pairwise tests, corrected using Holm’s method, revealed that human infants were rated as cuter if their eyes were visible (*t*(836) = -9.12, *p* < .001), and that puppy dogs were rated as *less* cute if their eyes were visible (*t*(836) = 2.45, *p* = .014).

Second, we examined ratings of vulnerability. Targets were rated as more vulnerable if their eyes were visible (β = 0.04, *t*(833) = 2.83, *p* < .001, 95% CI [0.01, 0.07]), and human infants were rated as more vulnerable than puppy dogs (β = -0.23, *t*(190) = -4.51, *p* < .001, 95% CI [-0.33, -0.13]). There was no statistically interaction (β = 0.05, *t*(833) = 0.81, *p* = .414, 95% CI [-0.01, 0.04]).

Third, we examined ratings of how much participants felt a need to protect a target. Participants reported a stronger need to protect targets if their eyes were visible (β = 0.02, *t*(833) = 1.97, *p* = .048, 95% CI [0.0002, 0.05]). They also reported a stronger need to protect human infants, compared to puppy dogs (β = -0.11, *t*(180) = -2.07, *p* = .039, 95% CI [-0.22, -0.006]). There was no statistically significant interaction (β = -0.01, *t*(833) = 0.82, *p* = .441, 95% CI [-0.03, 0.01]).

#### Analyses testing for moderating effects of PCAT

To test whether any effects of the eye visibility manipulation were moderated by PCAT, we used the same analytic strategy employed in Experiments 1 and 2 (for all the output from the models, see S12, S13 and S14 Tables). Results revealed three statistically significant interactions. On ratings of cuteness, there was a 3-way interaction between PCAT-Nurturance, target type, and eye visibility (β = -0.17, *t*(835) = -2.48, *p* = .013, 95% CI [-0.30, -0.03]), indicating that the 2-way interaction between target type and eye visibility (described above) was stronger among participants with higher-scores on PCAT-Nurturance. Also on ratings of cuteness, there was a 3-way interaction between PCAT-Protection, target type, and eye visibility on ratings of cuteness (β = 0.21, *t*(839) = 2.58, *p* = .009, 95% CI [0.05, 0.37]), indicating that PCAT-Protection more strongly predicted the perceived cuteness of puppy dogs (compared to human infants), and that this effect occurred especially strongly when targets’ eyes were visible. Finally, on ratings of the need to protect targets, there was a 2-way interaction between PCAT-Nurturance and the eye visibility manipulation (β = -0.16, *t*(836) = -3.01, *p* = .002, 95% CI [-0.27, -0.05]): The effect of eye visibility was stronger among individuals who scored lower on PCAT-Nurturance.

#### Exploratory analyses testing for moderating effects of demographic variables

Analyses testing for moderating effects of demographic variables revealed four statistically significant interactions involving the eye visibility manipulation. For ratings of cuteness, there was a 2-way interaction between the eye visibility manipulation and dog ownership (*p* = .003): The effect of the manipulation on ratings of targets’ cuteness was stronger among dog-owners (*p* < .001) compared to non-dog-owners (*p* = .061). For ratings of vulnerability, there was also an interaction between the eye visibility manipulation and dog ownership (*p* = .038): Dog-owners rated targets as more vulnerable when their eyes were visible vs. concealed (*p* = .001), but for non-dog-owners the effect of the manipulation was negligible (*p* = .632). For ratings of the need to protect, there was again an interaction between the eye visibility manipulation and dog ownership (*p* = .005), but this 2-way interaction was qualified by a 3-way interaction between eye visibility, dog ownership, and target type (*p* = .020): Dog-owners indicated a stronger need to protect puppy dogs when their eyes were visible (*p* = .011); in contrast, the eye visibility manipulation did not affect dog-owners’ ratings of human infants (*p* = .384), nor did it affect non-dog-owners’ ratings of either puppy dogs (*p* = .365) or human infants (*p* = .584). Full details are presented in supplementary material (see S1 Text).

### Summary of primary results from Experiment 3

As in Experiments 1 and 2, results revealed that human infants were perceived to be cuter when their eyes were visible (and other babyish features were not), compared to conditions in which other babyish features were visible (and eyes were not). Additionally, human infants were perceived to be more vulnerable and in need of protection when their eyes were visible. These latter effects also occurred in appraisals of puppy dogs, but the effect of eye visibility on perceived cuteness did not (in fact—in direct contrast to the results of Experiment 2—puppy dogs were rated to be less cute with their eyes were visible). There was some additional evidence indicating that effects of eye visibility on appraisals may be moderated by individual differences in parental care motives; but the specific moderating effects observed in Experiment 3 differed from those observed in Experiments 1 and 2.

### Discussion of results from Experiments 1–3

**Fig 2:**
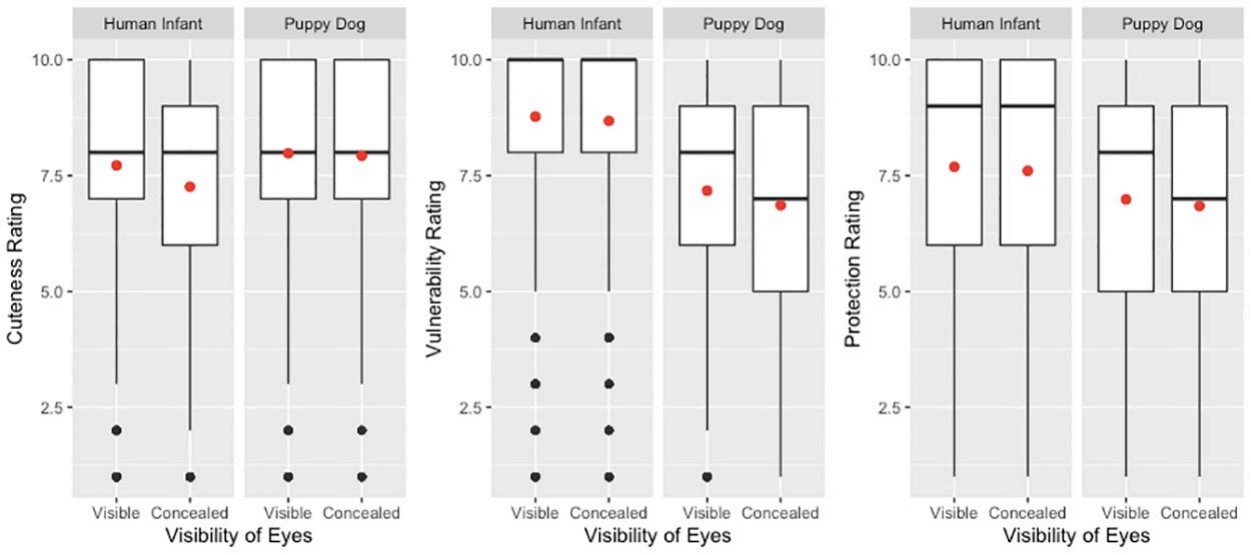
Experiments 1–3: Boxplots depicting ratings of human infants and puppy dogs, as a function of whether their eyes were visible or concealed. The boxplots describe data combined across all three experiments. Boxes indicate interquartile ranges. Bold horizontal lines indicate medians. Red dots indicated means. Black dots indicate outliers.

Are subjective appraisals of human infants influenced especially strongly by their eyes? The answer appears to be yes. Across 3 experiments (employing complementary methodologies) there emerged consistent evidence that human infants were perceived to be cuter when their eyes were visible (and other babyish features were not visible), compared to conditions in which other babyish features were visible (but eyes were not). There was also evidence—which emerged somewhat less consistently across studies—that when perceivers could see human infants’ eyes (compared to other babyish features), perceivers judged those human infants to be more vulnerable and felt a stronger need to protect them. (See Fig 2 for a graphical depiction of the effects that the eye visibility manipulation had, integrated across the 3 experiments).

Are there effects of eye visibility on appraisals of puppy dogs? The answer appears to be both yes and no. In 2 of the 3 experiments, there was evidence that when perceivers could see puppy dogs’ eyes (compared to other babyish features), they judged those puppy dogs to be more vulnerable and felt a stronger need to protect them. But there was no consistent evidence that eye visibility led to increased perceptions of puppy dogs’ cuteness (indeed, one experiment showed exactly the opposite effect).

Are these effects moderated by individual differences in parental care motives? The answer remains unclear. Each of the three experiments produced some evidence of moderating effects, but none of these effects emerged consistently across all studies.

Although the 3 experiments provide evidence that subjective appraisals of human infants and puppy dogs are influenced especially strongly by the their eyes, it remains unclear exactly why this is so. Given previous evidence that eye gaze affects several other kinds of appraisals [14, 21–24,32]—and because gaze direction can only be perceived when eyes are visible—we designed Experiments 4 and 5 to test whether appraisals of human infants and puppy dogs were influenced by eye contact.

## Experiment 4

### Methods

#### Participants

Participants were 287 adults residing in the United States (*M* age = 34.31 years; 116 women; 100 parents; 127 dog-owners) who were recruited on Mechanical Turk. Participants were randomly assigned to view photographs depicting either human infants (*n* = 145) or puppy dogs (*n* = 142).

#### Appraisals of human infants/puppy dogs

**Fig 3:**
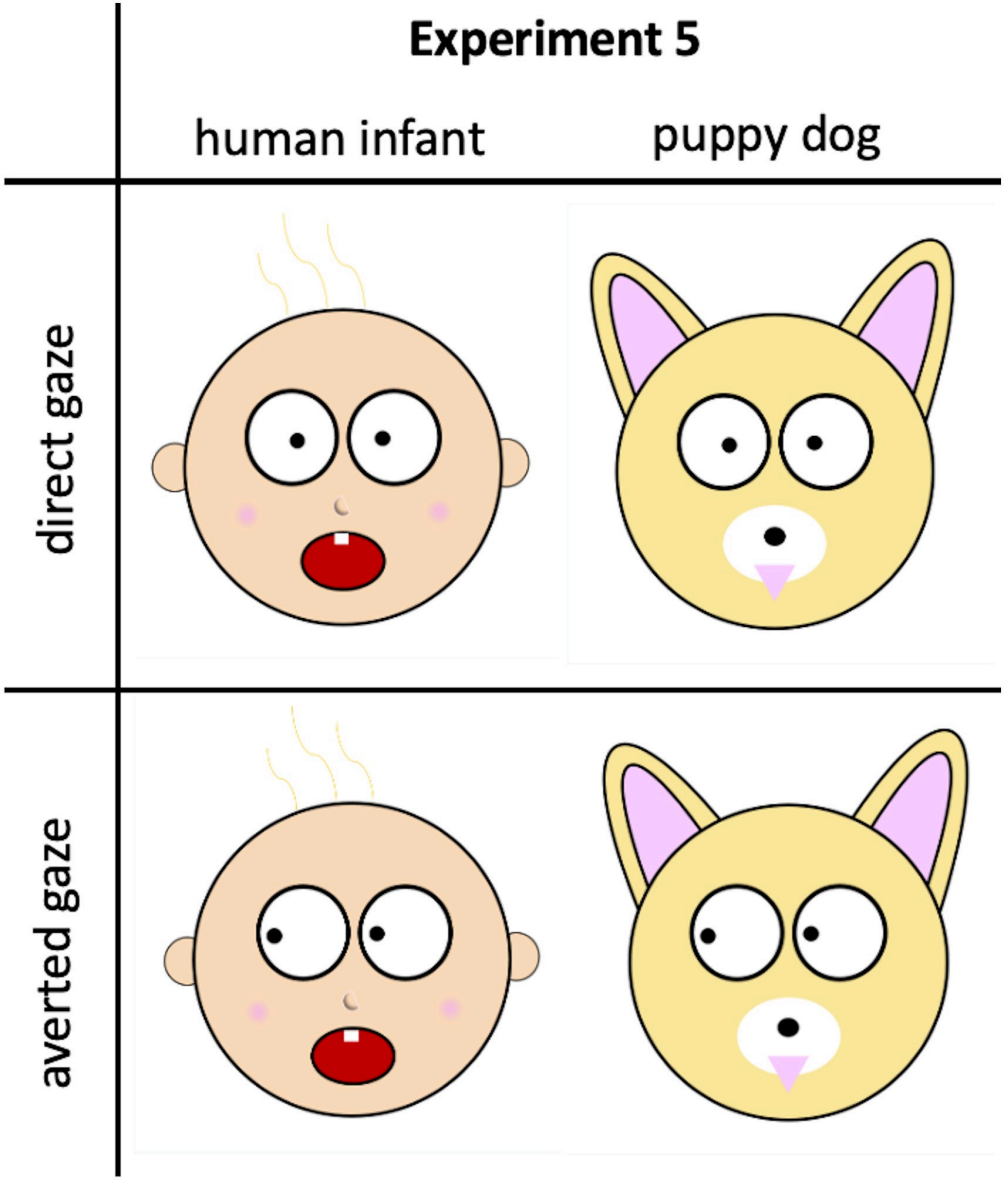
Example stimuli from Experiment 5. We are unable to share examples of the stimuli from Experiment 4 because of copyright issues.

Participants were presented serially with four photographs depicting either human infants or puppy dogs. (The original photographs were a subset of those of used in Experiments 1 and 2.) For two images, the photograph was retouched so the eyes gazed directly at the viewer. For the other two images, the photograph was retouched so the eyes gazed to the side (i.e., not at the viewer). All photographs were subsequently run through a pixilation filter (to reduce the possibility that retouched eyes might appear uncanny). Fig 3 depicts examples of stimuli that are similar to the stimuli that participants saw. Other than the manipulation that was imposed upon these images, all participants in the human infants condition saw the same images and all participants in the puppy dogs condition saw the same images. Across participants, we counterbalanced the images that had direct/averted eye gaze, and also counterbalanced order of presentation. Participants rated each human infant/puppy dog on the 3 rating measures employed in Experiment 3, assessing appraisals of “how cute” it was, “how vulnerable” it was, and how much they felt “a need to protect” it.

The effectiveness of the eye gaze manipulation was assessed on responses obtained from eleven additional participants, each of whom was presented with each stimulus image and asked where each target individual (human infant or puppy dog) was looking. Within this sample of 176 responses, 100% of responses to images of human infants and 98.9% of responses to images of puppy dogs indicated that the target individual was perceived to be looking in the direction intended (i.e., directly at the viewer, or away). To ascertain whether retouched (and pixilation-filtered) photographs still appeared realistic (and not uncanny), these same participants also rated each image according to “how normal” the target individual looked (1 = *not at all*; 5 = *extremely*). Stimulus images were chosen only if the mean rating was 3.5 or above.

#### Individual differences in parental care and tenderness

Participants completed the same individual difference measures as in Experiment 1. We computed participants’ scores on PCAT-Nurturance and PCAT-Protection (Cronbach’s αs = .91 and .90, respectively).

### Results

#### Effects of eye gaze manipulation on appraisals of human infants/puppy dogs

Table 4 summarizes mean ratings of human infants and puppy dogs, depending on direction of eye gaze. We conducted mixed-effects models on these ratings (for all output, see S15 Table). Fixed effects were the eye gaze manipulation (-0.5 = direct gaze, 0.5 = averted gaze), target type (-0.5 = human infant, 0.5 = puppy dog), and the interaction between these two variables. Random effects were participants and stimulus images.

**Table 4:** Experiment 4: Mean ratings (with standard deviations in parentheses) of human infants and puppy dogs, as a function of whether their eye gaze was direct or averted.

	Ratings of Human Infants		Ratings of Puppy Dogs	
	Direct Gaze	Averted Gaze	Direct Gaze	Averted Gaze
Cuteness	7.21	7.39	7.71	7.26
	(2.49)	(2.41)	(1.84)	(2.14)
Vulnerability	8.74	8.70	6.86	6.82
	(1.63)	(1.64)	(2.12)	(2.10)
Protection	7.28	7.30	6.47	6.19
	(2.81)	(2.81)	(2.60)	(2.73)

First, we examined ratings of cuteness. Targets were rated as cuter if their eye gaze was direct rather than averted (β = -0.13, *t*(854) = -2.24, *p* = .025, 95% CI [-0.05, -0.003]). The main effect for target type (human infants vs. puppy dogs) was non-significant (β = 0.18, *t*(81) = 0.64, *p* = .501, 95% CI [-0.07, 0.16]), but there was a significant interaction between target type and eye gaze (β = -0.63, *t*(854) = -5.34, *p* < .001, 95% CI [-0.09, -0.04]). Post-hoc pairwise tests, corrected using Holm’s method, revealed that human infants were rated as cuter if their gaze was averted (*t*(857) = -2.20, *p* = .027), and that puppy dogs were rated as cuter if their gaze was direct (*t*(857) = 5.32, *p* < .001).

Second, we examined ratings of vulnerability. Human infants were rated as more vulnerable than puppy dogs (β = -0.44, *t*(51) = -7.75, *p* < .001, 95% CI [-0.56, -0.33]). There was no statistically significant effect of the eye gaze manipulation (β = -0.009, *t*(854) = -0.73, *p* = .464, 95% CI [-0.03, 0.01]), nor was there an interaction (β = 0.00, *t*(854) = 0.04, *p* = .966, 95% CI [-0.02, 0.02]).

Third, we examined ratings of how much participants felt a need to protect a target. Participants reported a stronger need to protect targets when targets’ eye gaze was direct rather than averted (β = -0.02, *t*(854) = -2.50, *p* = .013, 95% CI [-0.04, -0.004]), and also reported a stronger need to protect human infants, compared to puppy dogs (β = -0.17, *t*(112) = -2.79, *p* = .006, 95% CI [-0.29, -0.05]). There was also a statistically significant interaction (β = -0.02, *t*(854) = -2.86, *p* = .004, 95% CI [-0.04, -0.008]). Post-hoc pairwise tests, corrected using Holm’s method, revealed that participants felt a stronger need to protect puppy dogs when puppy dogs’ eye gaze was direct (*t*(857) = 3.77, *p* < .001), but the eye gaze manipulation had no statistically significant effect on need to protect human infants (*t*(857) = -0.26, *p* = 0.794).

#### Analyses testing for moderating effects of PCAT

To test whether any of the preceding effects were moderated by PCAT, we conducted additional mixed-effects models that, in addition to the fixed effects and random effects identified above, also included the following fixed effects: The two PCAT subscales (PCAT-nurturance, PCAT-protection), as well as the two-way and three-way interactions between each subscale and the target type and eye gaze manipulations. Results revealed no statistically significant interactions between the eye gaze manipulation and either PCAT subscale (for all the output from the models, see S16, S17 and S18 Tables).

#### Exploratory analyses testing for moderating effects of demographic variables

Analyses testing for moderating effects of demographic variables revealed only one statistically significant interaction involving the gaze direction manipulation. For ratings of cuteness, there was a 3-way interaction between gaze direction manipulation, target type, and dog ownership (*p* = .020): Non-dog-owners rated puppy dogs as being cuter when gaze was direct vs. averted (*p* < .001) and human infants as being *less* cute when gaze was direct vs. averted (*p* = .029); in contrast; the gaze manipulation did not affect dog-owners’ ratings of either puppy dogs (*p* = .121) or human infants (*p* = 1.00). Full details are presented in supplementary material (see S1 Text).

### Summary of primary results from Experiment 4

Experiment 4 tested whether eye contact might amplify “parental” appraisals of human infants. Results provide no evidence of any such effect. (In fact, human infants were rated as *less* cute when they gazed directly at the viewer, compared to images in which their gaze was averted.) In contrast, there was some evidence that eye contact affects appraisals of puppy dogs: Compared to images in which puppy dogs gaze was averted, when puppy dogs gazed directly at the viewer they were rated to be cuter and also elicited a stronger need to offer protection. There was no evidence that any of these effects (or non-effects) were moderated by individual differences in inclinations toward nurturance or protection.

## Experiment 5

Experiment 5 provided a conceptual replication of Experiment 4. The experimental design was identical, but rather than employing photographic stimuli, participants in Experiment 5 were presented with cartoon drawings of human infants’ and puppy dogs’ faces. The direct of eye gaze (direct or averted) was manipulated within these cartoon images.

### Methods

#### Participants

Participants were 284 adults residing in the United States (*M* age = 37.98 years; 164 women, 3 other sex; 145 parents; 139 dog-owners) who were recruited on Mechanical Turk. Participants were randomly assigned to view cartoon images depicting either human infants (*n* = 142) or puppy dogs (*n* = 142).

#### Appraisals of human infants/puppy dogs

Participants were presented serially with four cartoon images depicting either human infants or puppy dogs. For two images, the image was created so the eyes gazed directly at the viewer. For the other two images, the image was created so the eyes gazed to the side (i.e., not at the viewer). The original cartoon images were identical to those of Study 3. Fig 3 depicts examples of stimuli. Other than the manipulation that was imposed upon these images, all participants in the human infants condition saw the same images and all participants in the puppy dogs condition saw the same images. Across participants, we counterbalanced the images that had direct/averted eye gaze, and also counterbalanced order of presentation. Across participants, we counterbalanced the specific images for which eye gaze was direct or averted. (Images were the same for all participants within each experimental condition; counterbalancing affected only which specific images included direct/averted gaze.) Also counterbalanced was order of image presentation. Participants rated each human infant/puppy dog on the same rating measures employed in Experiment 3.

#### Individual differences in parental care and tenderness

Participants completed the same individual difference measures as in Experiment 1. We computed participants’ scores on PCAT-Nurturance and PCAT-Protection (Cronbach’s αs = .92 and .86, respectively).

### Results

#### Effects of eye gaze manipulation on appraisals of human infants/puppy dogs

Table 5 summarizes mean ratings of human infants and puppy dogs, depending on direction of eye gaze. We conducted mixed-effects models identical to those of Experiment 4 (for all output, see S19 Table). On none of the rating measures were there statistically significant main effects of the eye gaze manipulation, nor were there statistically significant interactions of between the target type (human infant vs. puppy dog) and the eye gaze manipulation (absolute value of all βs ≤ 0.01; all *p*s ≥ .411).

**Table 5:** Mean ratings (with standard deviations in parentheses) of human infants and puppy dogs, as a function of whether their eye gaze was direct or averted.

	Ratings of Human Infants		Ratings of Puppy Dogs	
	Direct Gaze	Averted Gaze	Direct Gaze	Averted Gaze
Cuteness	5.21	5.16	6.46	6.47
	(2.66)	(2.67)	(2.36)	(2.30)
Vulnerability	7.07	6.98	5.53	5.54
	(2.42)	(2.32)	(2.54)	(2.46)
Protection	5.99	5.96	5.25	5.26
	(2.76)	(2.75)	(2.72)	(2.75)

#### Analyses testing for moderating effects of PCAT

To test whether any effects of the eye gaze manipulation might be moderated by PCAT, we used the same analytic strategy employed in Experiment 4 (for all the output from the models, see S20, S21 and S22 Tables). Results revealed just one such moderating effect: On ratings of cuteness, there was a statistically significant 2-way interaction between the eye gaze manipulation and the PCAT-Protection subscale (β = 0.13, *t*(848) = 2.13, *p* = .033, 95% CI [0.01, 0.26]): A positive effect of direct gaze (compared to averted gaze) on ratings of cuteness emerged among individuals with higher scores on PCAT-Protection.

#### Exploratory analyses testing for moderating effects of demographic variables

Analyses testing for moderating effects of demographic variables revealed two statistically significant 2-way interactions involving the gaze direction manipulation. For ratings of vulnerability, there was an interaction between the gaze direction manipulation and dog ownership (*p* = .003): non-dog-owners rated targets as being more vulnerable when gaze was direct vs. averted (*p* = .011), whereas the manipulation had a non-significant effect in the opposite direction among dog-owners (*p* = .097). For ratings of the need to protect, there was also an interaction between gaze aversion and dog ownership (*p* = .048); however, after correcting for multiple comparisons, for neither dog-owners nor non-dog-owners was there a significant effect of the manipulation (*p*s < .08). Full details are presented in supplementary material (see S1 Text).

### Summary of primary results from Experiment 5

Consistent with the results of Experiment 4, the results of Experiment 5 provided no evidence whatsoever that human infants elicited more “parental” appraisals if they gazed at, rather than away from, perceivers. There was also no evidence that eye gaze affected perceivers’ appraisals of puppy dogs. There was some evidence that eye gaze may have effects on appraisals of cuteness (of both human infants and puppy dogs) among individuals who have an especially strong inclination toward “parental” protectiveness.

### Discussion of results from Experiments 4–5

**Fig 4:**
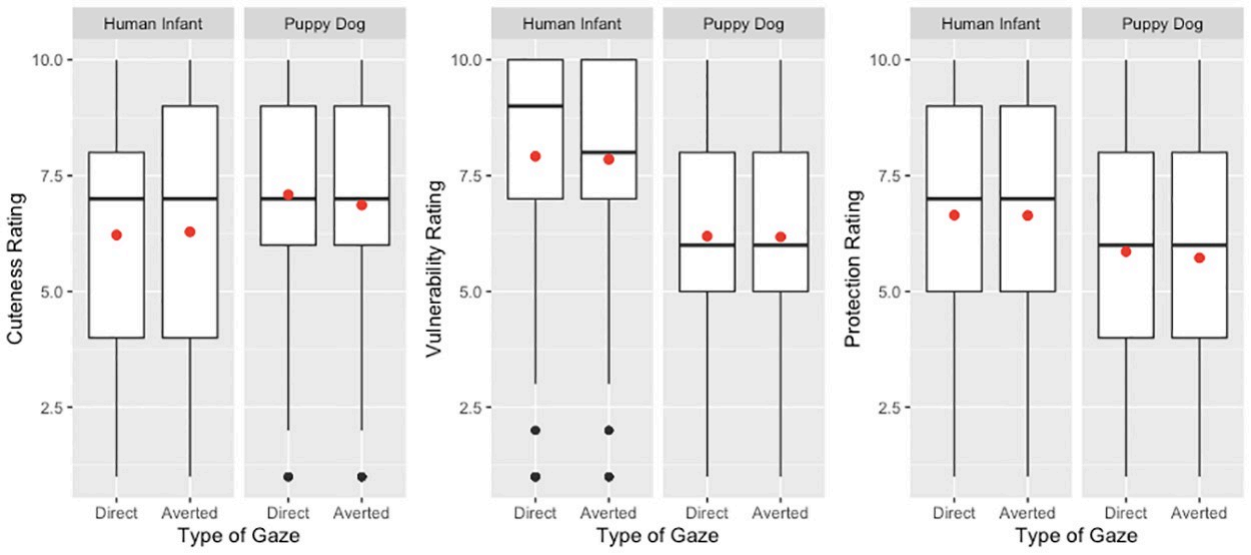
Experiments 4 and 5: Boxplots depicting ratings of human infants and puppy dogs, as a function of whether their eye gaze was direct or averted in Experiments 4 to 5. The boxplots describe data combined across both experiments. Boxes indicate interquartile ranges. Bold horizontal lines indicate medians. Red dots indicated means. Black dots indicate outliers.

If perceptual access to children’s eyes plays a special role in promoting “parental” appraisals of them (as suggested by the results of Experiments 1–3), might those effects occur specifically because it allows for the possibility of eye contact? If so, then one would expect that young children gazing directly at a perceiver (rather than gazing to the side) would also elicit more “parental” appraisals. Across two experiments, there was no evidence that this was the case. (See Fig 4 for a graphical depiction of the effects that the eye gaze manipulation had, integrated across the 2 experiments.) Intriguingly, Experiment 4 yielded some evidence that puppy dogs—but not human infants—were more likely to elicit such appraisals when they looked directly at perceivers. This effect complements previous findings showing that dog-owners affiliative feelings toward their dogs are enhanced by eye contact [33]. But this effect did not replicate in Study 5. Additionally, Experiment 5 yielded some evidence that eye gaze affected appraisals more among individuals who felt more strongly protective of children; but, given that no such effect was observed in Experiment 4, it appears premature to draw any meaningful conclusion from that one result. Given the lack of consistent evidence that actual eye contact meaningfully affects “parental” appraisals of human infants (or puppy dogs), the opportunity for eye contact does not appear to offer a compelling explanation for the finding (observed in Experiments 1–3) that the perception of human infants’ eyes contributes especially strongly to appraisals of their cuteness, vulnerability, and need for protection.

## General discussion

At the outset of this article we identified four main research questions, and the results obtained from five experiments provide answers to these questions. The following paragraphs provide a summary of the empirical answers to those questions, and their implications.

Are “parental” appraisals of young children influenced especially strongly by the visual perception of their eyes? The answer—obtained across 3 experiments—appears to be yes. Compared to control conditions (that provided perceptual access to other babyish features instead), when perceivers had perceptual access to human infants’ eyes, they perceived those infants to be cuter. There was also some evidence—which was less consistent across studies—that they also perceived those infants to be more vulnerable and in need of protection. Of course, it will be useful for future work to further replicate this result, ideally with different stimuli, to assure that these effects are not idiosyncratic to the specific stimuli employed in Experiments 1–3. It would also be useful to conduct replications that include additional control conditions too. Although previous research has linked appraisals of cuteness to the physical dimensions of eyes and other babyish features [11,13], the present findings reveal that, even compared to other facial babyish features, eyes play an outsized role in influencing the kinds of appraisals that promote caregiving responses to young children.

Is this effect attributable to eye contact? Results from 2 additional experiments indicate that the answer is no—or, at least, these results provided no evidence to compel a more affirmative answer. These null results cannot easily be attributed to floor or ceiling effects (results summarized in Tables 4 and 5 reveal ample variability on the variables of interest), nor to a failure of the eye gaze manipulation (as indicated by results on a manipulation check). These null results are interesting, given that eye contact does amplify appraisals and judgments in other domains of person perception [23–24, 32,34]. It remains unclear why no analogous effect emerged in this particular domain. Regardless, if indeed human infants’ eyes are an especially influential feature within the broader set of babyish features (as indicated by the results of Experiments 1–3), we suspect that is not because of what those eyes look at, but is instead because of what those eyes look like. The particular appearance of a person’s eyes is instrumental in conveying specific kinds of information that are relevant to parental caregiving, such as fatigue and sickness [35,36]. Adults might be especially vigilant for these kinds of clues in the faces of preverbal children, who lack the linguistic capability to communicate their needs.

Do the visual perception of eyes and eye contact influence appraisals of non-human animals? Our experiments focused on puppy dogs and—consistent with other research documenting both similarities and differences in adults’ responses to children and to dogs [37]—produced an answer that is not easily boiled down to a simple yes or no. Two experiments produced evidence that “parental” appraisals of puppy dogs are influenced especially strongly by the perception of puppy dogs’ eyes. But this effect was limited to appraisals of puppy dogs’ vulnerability and need for protection; in contrast to the positive effect of eye-visibility on appraisals of human infants’ cuteness, there was no analogous effect on appraisals of puppy dogs’ cuteness. The similar effects (on appraisals of vulnerability and need for protection) may reflect an overgeneralization effect of the same sort that leads people to judge baby-faced adults to be less capable than mature-faced adults [27]. The different effects (on appraisals of cuteness) might simply reflect idiosyncratic differences in the small samples of stimuli that were used in these studies; therefore, before drawing any confident conclusions based on these differences, it will be important for future studies to determine whether these differences also emerge when using additional sets of stimuli that, ideally, might be more representative of the entire populations of human infants and puppy dogs. If indeed these differences do replicate in future studies, they might plausibly reflect the functional different relationships that adults have with house pets [38–39] and with children. The former is primarily associated with caregiving behaviors that help pets to survive; whereas the latter is associated with many additional forms of caregiving behavior that help children not merely to survive but also to thrive (e.g., to succeed in academic pursuits and social relationships). Subjective appraisals of cuteness may tacitly connote potential to thrive and may elicit behavioral inclinations accordingly [20], and so may be more functionally relevant to adults’ relationships with children than to their relationships with dogs.

Are these effects moderated by individual differences in the protective and nurturant responses that characterize the parental care motivational system? Again, the results cannot compel a simple yes or no answer. Four of the 5 experiments yielded some evidence that these individual differences (assessed by the PCAT questionnaire) may moderate effects of eye visibility and/or eye contact; but the specific effects differed, and none of these specific moderating effects replicated across multiple studies.

Further analyses of PCAT scores revealed additional findings that—although ancillary to the four main research questions identified above—did replicate across studies and may have implications for understanding differences in adults’ responses to human infants and puppy dogs. These findings pertained to the unique predictive utility of the “protection” and “nurturance” subscales of the PCAT questionnaire. In experiments that employed photographs as stimuli (but not in experiments that employed cartoons as stimuli), individual differences in parental protectiveness more strongly predicted appraisals of puppy dogs than appraisals of human infants, whereas individual differences in parental nurturance more strongly predicted appraisals of human infants than appraisals of puppy dogs. These different patterns of association—like some of the other effects summarized above—likely reflect differences in the functional relationships that people typically have with pets and children. These novel findings extend previous results documenting different implications associated with motivational inclinations toward parental protectiveness and nurturance [30], and highlight the utility of conceptually distinguishing between—and measuring—these two underlying facets of the parental care motivational system.

Collectively, these findings contribute to the psychological literature attesting to the importance of eyes in the domain of person perception and social inference [14]. In particular, these results provide evidence that the perception of eyes is especially influential—even more influential than other facial features—in eliciting prototypically “parental” appraisals of young children. The dependent measures in these studies were limited to appraisals (e.g., subjective rating of cuteness and vulnerability) and, in future research, it would be informative to assess whether similar effects might be obtained on additional responses that may be associated with these appraisals (e.g., visual attention) and on actual caregiving behaviors (including both protective and nurturant behaviors). Additional applications to care-giving behavior might be worth exploring too—such as the implied possibility that people who are generally more attentive to other’s eyes might generally perceive children to be cuter and, consequently, to respond to children in a more caring way.

Additional results indicated that the perception of eyes may also be especially influential in eliciting specific kinds of appraisals of puppy dogs too, and these findings too might be fruitfully followed up in studies that focus on other kinds of outcomes. For instance, people find baby animals—compared to adult animals—to be less appetizing as sources of meat [40]. Might the size of this effect depend on the extent to which those animal’s eyes are perceptible? A different line of research reveals that the presence of dogs and other house pets can reduce humans’ experience of stress [41]. Might this stress-buffering effect also be moderated by visual access to those animals’ eyes? More generally, if indeed eyes have an outsized effect on “parental” responses to human infants and puppy dogs, there are a wide range of potential implications that may merit closer attention.

## Supporting information

S1 Table Mixed-effects models for effects of eye visibility and target type on ratings in Experiment 1. (DOCX)

S2 Table Mixed-effects models for moderating effects of parental care and tenderness on cuteness in Experiment 1. (DOCX)

S3 Table Mixed-effects models for moderating effects of parental care and tenderness on vulnerability in Experiment 1. (DOCX)

S4 Table Mixed-effects models for moderating effects of parental care and tenderness on self-reliance in Experiment 1. (DOCX)

S5 Table Mixed-effects models for moderating effects of parental care and tenderness on need to protect in Experiment 1. (DOCX)

S6 Table Mixed-Effects models for effects of eye visibility and target type on ratings in Experiment 2. (DOCX)

S7 Table Mixed-effects models for moderating effects of parental care and tenderness on cuteness in Experiment 2. (DOCX)

S8 Table Mixed-effects models for moderating effects of parental care and tenderness on vulnerability in Experiment 2. (DOCX)

S9 Table Mixed-effects models for moderating effects of parental care and tenderness on self-reliance in Experiment 2. (DOCX)

S10 Table Mixed-effects models for moderating effects of parental care and tenderness on need to protect in Experiment 2. (DOCX)

S11 Table mixed-effects models for effects of eye visibility and target type on ratings in Experiment 3. (DOCX)

S12 Table Mixed-effects models for moderating effects of parental care and tenderness on cuteness in Experiment 3. (DOCX)

S13 Table Mixed-effects models for moderating effects of parental care and tenderness on vulnerability in Experiment 3. (DOCX)

S14 Table Mixed-effects models for moderating effects of parental care and tenderness on need to protect in Experiment 3. (DOCX)

S15 Table Mixed-effects models for effects of gaze aversion and target type on ratings in Experiment 4. (DOCX)

S16 Table Mixed-effects models for moderating effects of parental care and tenderness on cuteness in Experiment 4. (DOCX)

S17 Table Mixed-effects models for moderating effects of parental care and tenderness on vulnerability in Experiment 4. (DOCX)

S18 Table Mixed-effects models for moderating effects of parental care and tenderness on need to protect in Experiment 4. (DOCX)

S19 Table Mixed-effects models for effects of gaze aversion and target type on ratings in Experiment 5. (DOCX)

S20 Table Mixed-effects models for moderating effects of parental care and tenderness on cuteness in Experiment 5. (DOCX)

S21 Table Mixed-effects models for moderating effects of parental care and tenderness on vulnerability in Experiment 5. (DOCX)

S22 Table Mixed-effects models for moderating effects of parental care and tenderness on need to protect in Experiment 5. (DOCX)

S1 Text Analyses testing for moderating effects of gender, parental status, and dog ownership. (DOCX)

## References

[1] Rilling JK. The neural and hormonal bases of human parental care. Neuropsychologia. 2013 3 1;51(4):731–47. 10.1016/j.neuropsychologia.2012.12.017 23333868

[2] Bos PA. The endocrinology of human caregiving and its intergenerational transmission. Dev Psychopathol. 2017 8;29(3):971–999. 10.1017/S0954579416000973 27760577

[3] Schaller M. The parental care motivational system and why it matters (for everyone). Curr Dir Psychol Sci. 2018 10;27(5):295–301.

[4] Glocker ML, Langleben DD, Ruparel K, Loughead JW, Valdez JN, Griffin MD, et al Baby schema modulates the brain reward system in nulliparous women. Proc Natl Acad Sci U S A. 2009 6 2;106(22):9115–9. 10.1073/pnas.0811620106 19451625 PMC2690007

[5] Bos PA, Spencer H, Montoya ER. Oxytocin reduces neural activation in response to infant faces in nulliparous young women. Social cognitive and affective neuroscience. 2018 10;13(10):1099–109. 10.1093/scan/nsy080 30203082 PMC6204485

[6] Lorenz K. The innate forms of possible experience. [*Die angeborenen formen möglicher erfahrung*] Ethology. [*Zeitschrift für Tierpsychologie*] 1943 1 12;5(2):235–409.

[7] Glocker ML, Langleben DD, Ruparel K, Loughead JW, Gur RC, Sachser N. Baby schema in infant faces induces cuteness perception and motivation for caretaking in adults. Ethology. 2009 3;115(3):257–63. 10.1111/j.1439-0310.2008.01603.x 22267884 PMC3260535

[8] Kringelbach ML, Stark EA, Alexander C, Bornstein MH, Stein A. On cuteness: Unlocking the parental brain and beyond. Trends Cogn Sci. 2016 7 1;20(7):545–58. 10.1016/j.tics.2016.05.003 27211583 PMC4956347

[9] Lobmaier JS, Sprengelmeyer R, Wiffen B, Perrett DI. Female and male responses to cuteness, age and emotion in infant faces. Evolution and Human Behavior. 2010 1 1;31(1):16–21.

[10] Sherman GD, Haidt J, Coan JA. Viewing cute images increases behavioral carefulness. Emotion. 2009 4;9(2):282–6. 10.1037/a0014904 19348541

[11] Hildebrandt KA, Fitzgerald HE. Facial feature determinants of perceived infant attractiveness. Infant Behav Dev. 1979 1 1;2:329–39.

[12] Little AC. Manipulation of infant-like traits affects perceived cuteness of infant, adult and cat faces. Ethology. 2012 8;118(8):775–82.

[13] Sternglanz SH, Gray JL, Murakami M. Adult preferences for infantile facial features: An ethological approach. Animal Behav. 1977 2 1;25:108–15. 10.1016/0003-3472(77)90072-0 855947

[14] Grossmann T. The eyes as windows into other minds: An integrative perspective. Perspect Psychol Sci. 2017 1;12(1):107–21. 10.1177/1745691616654457 28073330

[15] Borgi M, Cogliati-Dezza I, Brelsford V, Meints K, Cirulli F. Baby schema in human and animal faces induces cuteness perception and gaze allocation in children. Front Psychol. 2014 5 7;5:411 10.3389/fpsyg.2014.00411 24847305 PMC4019884

[16] Henderson JM, Williams CC, Falk RJ. Eye movements are functional during face learning. Mem Cognit. 2005 1 1;33(1):98–106. 10.3758/bf03195300 15915796

[17] Janik SW, Wellens AR, Goldberg ML, Dell'Osso LF. Eyes as the center of focus in the visual examination of human faces. Percept Motor Skills. 1978 12;47(3):857–8. 740480 10.2466/pms.1978.47.3.857

[18] Balas B, Horski J. You can take the eyes out of the doll, but…. Perception. 2012 3;41(3):361–4. 10.1068/p7166 22808589 PMC4041204

[19] Looser CE, Wheatley T. The tipping point of animacy: How, when, and where we perceive life in a face. Psychol Sci. 2010 12;21(12):1854–62. 10.1177/0956797610388044 21097720

[20] Sherman GD, Haidt J. Cuteness and disgust: The humanizing and dehumanizing effects of emotion. Emot Rev. 2011 7;3(3):245–51.

[21] Chen FS, Minson JA, Schöne M, Heinrichs M. In the eye of the beholder: Eye contact increases resistance to persuasion. Psychological Sci. 2013 11;24(11):2254–61. 10.1177/0956797613491968 24068114

[22] Emery NJ. The eyes have it: the neuroethology, function and evolution of social gaze. Neurosci Biobehav Rev. 2000 8 1;24(6):581–604. 10.1016/s0149-7634(00)00025-7 10940436

[23] Khalid S, Deska JC, Hugenberg K. The eyes are the windows to the mind: Direct eye gaze triggers the ascription of others’ minds. Pers Soc Psychol Bull. 2016 12;42(12):1666–77. 10.1177/0146167216669124 27738192

[24] Kellerman J, Lewis J, Laird JD. Looking and loving: The effects of mutual gaze on feelings of romantic love. J Res Pers. 1989 6 1;23(2):145–61.

[25] Kim S, Fonagy P, Koos O, Dorsett K, Strathearn L. Maternal oxytocin response predicts mother-to-infant gaze. Brain Res. 2014 9 11;1580:133–42. 10.1016/j.brainres.2013.10.050 24184574 PMC4286383

[26] Buckels EE, Beall AT, Hofer MK, Lin EY, Zhou Z, Schaller M. Individual differences in activation of the parental care motivational system: Assessment, prediction, and implications. J Pers Soc Psychol. 2015 3;108(3):497–514. 10.1037/pspp0000023 25559194

[27] Zebrowitz LA, Montepare JM. Social psychological face perception: Why appearance matters. Soc Pers Psychol Compass. 2008 5;2(3):1497–517 10.1111/j.1751-9004.2008.00109.x PMC2811283 20107613

[28] Brosch T, Sander D, Scherer KR. That baby caught my eye … attention capture by infant faces. Emotion. 2007 8, 7(3):685–89. 10.1037/1528-3542.7.3.685 17683225

[29] Golle J, Lisibach S, Mast FW, Lobmaier JS. Sweet puppies and cute babies: Perceptual adaptation to babyfacedness transfers across species. PLoS One. 2013 3 13;8(3):e58248 10.1371/journal.pone.0058248 23516453 PMC3596402

[30] Hofer MK, Buckels EE, White CJ, Beall AT, Schaller M. Individual differences in activation of the parental care motivational system: An empirical distinction between protection and nurturance. Soc Psychol Personal Sci. 2018 11;9(8):907–16.

[31] Hahn AC, DeBruine LM, Jones BC. Reported maternal tendencies predict the reward value of infant facial cuteness, but not cuteness detection. Biol Lett. 2015 3 31;11(3) 10.1098/rsbl.2014.0978 PMC4387492 25740842

[32] Macrae CN, Hood BM, Milne AB, Rowe AC, Mason MF. Are you looking at me? Eye gaze and person perception. Psychol Sci. 2002 9;13(5):460–4. 10.1111/1467-9280.00481 12219814

[33] Nagasawa M, Mitsui S, En S, Ohtani N, Ohta M, Sakuma Y, et al Oxytocin-gaze positive loop and the coevolution of human-dog bonds. Science. 2015 4 17;348(6232):333–6. 10.1126/science.1261022 25883356

[34] Adams RB Jr, Kleck RE. Effects of direct and averted gaze on the perception of facially communicated emotion. Emotion. 2005 3;5(1):3–11. 10.1037/1528-3542.5.1.3 15755215

[35] Sundelin T, Lekander M, Kecklund G, Van Someren EJ, Olsson A, Axelsson J. Cues of fatigue: effects of sleep deprivation on facial appearance. Sleep 2013 36: 1355–60. 10.5665/sleep.2964 23997369 PMC3738045

[36] Axelsson J, Sundelin T, Olsson MJ, Sorjonen K, Axelsson C, Lasselin J, et al 2018 Identification of acutely sick people and facial cues of sickness. Proc Royal Soc B 2018 285: 20172430. 10.1098/rspb.2017.2430 PMC5784201 29298938

[37] Stoeckel LE, Palley LS, Gollub RL, Niemi SM, Evins AE. Patterns of brain activation when mothers view their own child and dog: An fMRI study. PLoS One. 2014 10 3;9(10):e107205 10.1371/journal.pone.0107205 25279788 PMC4184794

[38] Amiot CE, Bastian B. Toward a psychology of human–animal relations. Psychol Bull. 2015 1;141(1):6–47. 10.1037/a0038147 25365760

[39] Archer J. Why do people love their pets? Evol Psychol. 1997 7 1;18(4):237–59.

[40] Piazza J, McLatchie N, Olesen C. Are baby animals less appetizing? Tenderness toward baby animals and appetite for meat. Anthrozoos. 2018 5 4;31(3):319–35.

[41] McConnell AR, Brown CM, Shoda TM, Stayton LE, Martin CE. Friends with benefits: on the positive consequences of pet ownership. J Pers Soc Psychol. 2011 12;101(6):1239–52. 10.1037/a0024506 21728449

