# Supplementary material for: “Parental” responses to human infants (and puppy dogs): Evidence that the perception of eyes is especially influential, but eye contact is not
Source: PLoS One. 2020 May 6;15(5):e0232059. doi: 10.1371/journal.pone.0232059 (PMC7202593; doi:10.1371/journal.pone.0232059)
Supplement: S1 Table — (DOCX) [file pone.0232059.s001.docx]

**S1 Table. Mixed-Effects Models for Effects of Eye Visibility and Target Type on Ratings in Experiment 1.**

|  | β | *t* | *df*s | *p* | 95% CI |
| --- | --- | --- | --- | --- | --- |
| Cuteness |  |  |  |  |  |
| Eye Visibility | 0.05 | 7.13 | 2127 | < .001 | [0.04, 0.07] |
| Target Type | 0.21 | 3.96 | 254 | < .001 | [0.10, 0.32] |
| Interaction | -0.05 | -6.69 | 2127 | < .001 | [-0.07, -0.03] |
| Vulnerability |  |  |  |  |  |
| Eye Visibility | 0.03 | 3.55 | 2127 | < .001 | [0.01, 0.04] |
| Target Type | -0.34 | -7.09 | 297 | < .001 | [-0.44, -0.25] |
| Interaction | 0.00 | 0.42 | 2127 | .644 | [-0.01, 0.02] |
| Self-Reliance |  |  |  |  |  |
| Eye Visibility | 0.00 | 0.35 | 2127 | .725 | [-0.01, 0.01] |
| Target Type | 0.46 | 9.57 | 280 | < .001 | [0.37, 0.56] |
| Interaction | -0.01 | -1.42 | 2127 | .153 | [-0.02, 0.003] |
| Need to Protect |  |  |  |  |  |
| Eye Visibility | 0.01 | 1.86 | 2127 | .062 | [-0.0005, 0.02] |
| Target Type | -0.08 | -1.58 | 311 | .114 | [-0.19, 0.02] |
| Interaction | -0.007 | -1.25 | 2127 | .210 | [-0.01, 0.004] |
