# Supplementary material for: “Parental” responses to human infants (and puppy dogs): Evidence that the perception of eyes is especially influential, but eye contact is not
Source: PLoS One. 2020 May 6;15(5):e0232059. doi: 10.1371/journal.pone.0232059 (PMC7202593; doi:10.1371/journal.pone.0232059)
Supplement: S2 Table — (DOCX) [file pone.0232059.s002.docx]

**S2 Table. Mixed-Effects Model for Moderating Effects of Parental Care and Tenderness on Cuteness in Experiment 1.**

|  | β | *t* | *df*s | *p* | 95% CI |
| --- | --- | --- | --- | --- | --- |
| Eye Visibility | 0.07 | 1.85 | 2128 | .064 | [-0.004, 0.15] |
| Target Type | 1.37 | 7.42 | 310 | < .001 | [1.01, 1.73] |
| Nurturance | 0.39 | 8.16 | 305 | < .001 | [0.29, 0.48] |
| Protection | 0.12 | 2.65 | 305 | .008 | [0.03, 0.21] |
| Interaction of Visibility and Target Type | -0.05 | -1.35 | 2128 | .174 | [-0.13, 0.02] |
| Interaction of Visibility and Nurturance | -0.09 | -2.31 | 2127 | .020 | [-0.17, -0.01] |
| Interaction of Target Type and Nurturance | -1.42 | -7.63 | 305 | < .001 | [-1.78, -1.05] |
| Interaction of Visibility and Protection | 0.07 | 1.69 | 2127 | .089 | [-0.01, 0.16] |
| Interaction of Target Type and Protection | 0.24 | 1.13 | 305 | .257 | [-0.17, 0.66] |
| Interaction of Visibility, Type, and Nurturance | 0.03 | 0.83 | 2127 | .402 | [-0.04, 0.11] |
| Interaction of Visibility, Type, and Protection | -0.03 | -0.77 | 2127 | .436 | [-0.12, 0.05] |
