# Supplementary material for: “Parental” responses to human infants (and puppy dogs): Evidence that the perception of eyes is especially influential, but eye contact is not
Source: PLoS One. 2020 May 6;15(5):e0232059. doi: 10.1371/journal.pone.0232059 (PMC7202593; doi:10.1371/journal.pone.0232059)
Supplement: S3 Table — (DOCX) [file pone.0232059.s003.docx]

**S3 Table. Mixed-Effects Model for Moderating Effects of Parental Care and Tenderness on Vulnerability in Experiment 1.**

|  | β | *t* | *df*s | *p* | 95% CI |
| --- | --- | --- | --- | --- | --- |
| Eye Visibility | 0.05 | 1.41 | 2130 | .157 | [-0.02, 0.14] |
| Target Type | -0.31 | -1.40 | 306 | .162 | [-0.74, 0.12] |
| Nurturance | 0.11 | 2.03 | 305 | .042 | [0.004, 0.23] |
| Protection | 0.09 | 1.61 | 305 | .108 | [-0.02, 0.20] |
| Interaction of Visibility and Target Type | 0.02 | 0.58 | 2130 | .561 | [-0.05, 0.10] |
| Interaction of Visibility and Nurturance | -0.05 | -1.30 | 2127 | .191 | [-0.13, 0.02] |
| Interaction of Target Type and Nurturance | -0.36 | -1.63 | 305 | .103 | [-0.80, 0.07] |
| Interaction of Visibility and Protection | 0.02 | 0.53 | 2128 | .592 | [-0.06, 0.12] |
| Interaction of Target Type and Protection | 0.33 | 1.30 | 305 | .192 | [-0.16, 0.84] |
| Interaction of Visibility, Type, and Nurturance | -0.02 | -0.65 | 2127 | .510 | [-0.11, 0.05] |
| Interaction of Visibility, Type, and Protection | 0.00 | 0.13 | 2128 | .893 | [-0.08, 0.10] |
