# Supplementary material for: “Parental” responses to human infants (and puppy dogs): Evidence that the perception of eyes is especially influential, but eye contact is not
Source: PLoS One. 2020 May 6;15(5):e0232059. doi: 10.1371/journal.pone.0232059 (PMC7202593; doi:10.1371/journal.pone.0232059)
Supplement: S4 Table — (DOCX) [file pone.0232059.s004.docx]

**S4 Table. Mixed-Effects Model for Moderating Effects of Parental Care and Tenderness on Self-Reliance in Experiment 1.**

|  | β | *t* | *df*s | *p* | 95% CI |
| --- | --- | --- | --- | --- | --- |
| Eye Visibility | -0.02 | -0.80 | 2128 | .422 | [-0.09, 0.04] |
| Target Type | -0.57 | 2.57 | 308 | .010 | [0.13, 1.00] |
| Nurturance | -0.02 | -0.47 | 306 | .636 | [-0.13, 0.08] |
| Protection | 0.00 | 0.00 | 306 | .999 | [-0.11, 0.11] |
| Interaction of Visibility and Target Type | 0.01 | 0.32 | 2128 | .748 | [-0.05, 0.07] |
| Interaction of Visibility and Nurturance | 0.03 | 0.96 | 2127 | .336 | [-0.03, 0.10] |
| Interaction of Target Type and Nurturance | -0.22 | -0.99 | 306 | .322 | [-0.65, 0.21] |
| Interaction of Visibility and Protection | -0.0002 | -0.06 | 2127 | .949 | [-0.08, 0.07] |
| Interaction of Target Type and Protection | 0.11 | 0.43 | 306 | .662 | [-0.38, 0.61] |
| Interaction of Visibility, Type, and Nurturance | -0.04 | -1.37 | 2127 | .169 | [-0.11, 0.02] |
| Interaction of Visibility, Type, and Protection | 0.02 | 0.64 | 2127 | .517 | [-0.05, 0.10] |
