# Supplementary material for: “Parental” responses to human infants (and puppy dogs): Evidence that the perception of eyes is especially influential, but eye contact is not
Source: PLoS One. 2020 May 6;15(5):e0232059. doi: 10.1371/journal.pone.0232059 (PMC7202593; doi:10.1371/journal.pone.0232059)
Supplement: S5 Table — (DOCX) [file pone.0232059.s005.docx]

**S5 Table. Mixed-Effects Models for Moderating Effects of Parental Care and Tenderness on Need to Protect in Experiment 1.**

|  | β | *t* | *Df*s | *p* | 95% CI |
| --- | --- | --- | --- | --- | --- |
| Eye Visibility | 0.02 | 0.90 | 2129 | .364 | [-0.03, 0.08] |
| Target Type | 0.72 | 3.62 | 306 | < .001 | [0.33, 1.11] |
| Nurturance | 0.33 | 6.50 | 305 | < .001 | [0.23, 0.43] |
| Protection | 0.28 | 5.50 | 305 | < .001 | [0.18, 0.38] |
| Interaction of Visibility and Target Type | -0.01 | -0.37 | 2129 | .710 | [-0.06, 0.04] |
| Interaction of Visibility and Nurturance | 0.01 | 0.50 | 2127 | .613 | [-0.04, 0.07] |
| Interaction of Target Type and Nurturance | -1.14 | -5.65 | 305 | < .001 | [-1.53, -0.74] |
| Interaction of Visibility and Protection | -0.02 | 0.89 | 2127 | .372 | [-0.09, 0.03] |
| Interaction of Target Type and Protection | 0.32 | 1.41 | 305 | .159 | [-0.12, 0.77] |
| Interaction of Visibility, Type, and Nurturance | 0.00 | 0.14 | 2127 | .887 | [-0.05, 0.06] |
| Interaction of Visibility, Type, and Protection | -0.0009 | -0.03 | 2127 | .976 | [-0.06, 0.06] |
