# Supplementary material for: “Parental” responses to human infants (and puppy dogs): Evidence that the perception of eyes is especially influential, but eye contact is not
Source: PLoS One. 2020 May 6;15(5):e0232059. doi: 10.1371/journal.pone.0232059 (PMC7202593; doi:10.1371/journal.pone.0232059)
Supplement: S7 Table — (DOCX) [file pone.0232059.s007.docx]

**S7 Table. Mixed-Effects Model for Moderating Effects of Parental Care and Tenderness on Cuteness in Experiment 2.**

|  | β | *t* | *df*s | *p* | 95% CI |
| --- | --- | --- | --- | --- | --- |
| Eye Visibility | -0.09 | -1.76 | 2092 | .078 | [-0.19, 0.01] |
| Target Type | 0.46 | 2.38 | 308 | .017 | [0.08, 0.85] |
| Nurturance | 0.40 | 9.09 | 300 | < .001 | [0.31, 0.49] |
| Protection | 0.16 | 3.62 | 300 | < .001 | [0.07, 0.24] |
| Interaction of Visibility and Target Type | -0.04 | -0.90 | 2092 | .363 | [-0.14, 0.05] |
| Interaction of Visibility and Nurturance | -0.07 | -1.80 | 2092 | .071 | [-0.16, 0.006] |
| Interaction of Target Type and Nurturance | -0.98 | -5.99 | 300 | < .001 | [-1.31, -0.66] |
| Interaction of Visibility and Protection | 0.22 | 4.22 | 2092 | < .001 | [0.12, 0.33] |
| Interaction of Target Type and Protection | 0.52 | 2.58 | 300 | .010 | [0.12, 0.92] |
| Interaction of Visibility, Type, and Nurturance | -0.03 | 0.74 | 2092 | .459 | [-0.11, 0.05] |
| Interaction of Visibility, Type, and Protection | 0.08 | 1.62 | 2092 | .104 | [-0.01, 0.19] |
