# Supplementary material for: “Parental” responses to human infants (and puppy dogs): Evidence that the perception of eyes is especially influential, but eye contact is not
Source: PLoS One. 2020 May 6;15(5):e0232059. doi: 10.1371/journal.pone.0232059 (PMC7202593; doi:10.1371/journal.pone.0232059)
Supplement: S8 Table — (DOCX) [file pone.0232059.s008.docx]

**S8 Table. Mixed-Effects Model for Moderating Effects of Parental Care and Tenderness on Vulnerability in Experiment 2.**

|  | β | *t* | *Df*s | *p* | 95% CI |
| --- | --- | --- | --- | --- | --- |
| Eye Visibility | 0.06 | 1.44 | 2092 | .149 | [-0.02, 0.15] |
| Target Type | -0.98 | -5.18 | 307 | < .001 | [-1.35, -0.61] |
| Nurturance | 0.08 | 2.00 | 300 | .045 | [0.002, 0.17] |
| Protection | 0.16 | 3.88 | 300 | < .001 | [0.08, 0.25] |
| Interaction of Visibility and Target Type | 0.04 | 0.96 | 2092 | .332 | [-0.04, 0.13] |
| Interaction of Visibility and Nurturance | -0.02 | -0.55 | 2092 | .582 | [-0.09, 0.05] |
| Interaction of Target Type and Nurturance | -0.04 | -0.25 | 300 | .796 | [-0.35, 0.27] |
| Interaction of Visibility and Protection | 0.01 | 0.35 | 2092 | .726 | [-0.07, 0.11] |
| Interaction of Target Type and Protection | 0.49 | 2.53 | 300 | .011 | [0.11, 0.88] |
| Interaction of Visibility, Type, and Nurturance | -0.05 | -1.32 | 2092 | .186 | [-0.12, 0.02] |
| Interaction of Visibility, Type, and Protection | 0.05 | 1.19 | 2092 | .231 | [-0.03, 0.15] |
