# Supplementary material for: “Parental” responses to human infants (and puppy dogs): Evidence that the perception of eyes is especially influential, but eye contact is not
Source: PLoS One. 2020 May 6;15(5):e0232059. doi: 10.1371/journal.pone.0232059 (PMC7202593; doi:10.1371/journal.pone.0232059)
Supplement: S9 Table — (DOCX) [file pone.0232059.s009.docx]

**S9 Table. Mixed-Effects Model for Moderating Effects of Parental Care and Tenderness on Self-Reliance in Experiment 2.**

|  | β | *t* | *df*s | *p* | 95% CI |
| --- | --- | --- | --- | --- | --- |
| Eye Visibility | 0.02 | 0.69 | 2093 | .487 | [-0.04, 0.09] |
| Target Type | 0.71 | 3.67 | 304 | < .001 | [0.33, 1.09] |
| Nurturance | 0.07 | 1.77 | 301 | .077 | [-0.008, 0.16] |
| Protection | -0.08 | -1.87 | 301 | .061 | [-0.17, 0.003] |
| Interaction of Visibility and Target Type | -0.03 | -0.87 | 2093 | .382 | [-0.10, 0.03] |
| Interaction of Visibility and Nurturance | -0.03 | -1.06 | 2092 | .287 | [-0.09, 0.02] |
| Interaction of Target Type and Nurturance | -0.08 | -0.50 | 301 | .615 | [-0.40, 0.23] |
| Interaction of Visibility and Protection | -0.03 | -0.80 | 2093 | .424 | [-0.10, 0.04] |
| Interaction of Target Type and Protection | 0.00 | 0.01 | 301 | .990 | [-0.39, 0.39] |
| Interaction of Visibility, Type, and Nurturance | -0.03 | -1.12 | 2092 | .260 | [-0.09, 0.02] |
| Interaction of Visibility, Type, and Protection | 0.03 | 1.03 | 2093 | .300 | [-0.03, 0.11] |
