# Supplementary material for: “Parental” responses to human infants (and puppy dogs): Evidence that the perception of eyes is especially influential, but eye contact is not
Source: PLoS One. 2020 May 6;15(5):e0232059. doi: 10.1371/journal.pone.0232059 (PMC7202593; doi:10.1371/journal.pone.0232059)
Supplement: S10 Table — (DOCX) [file pone.0232059.s010.docx]

**S10 Table. Mixed-Effects Model for Moderating Effects of Parental Care and Tenderness on Need to Protect in Experiment 2.**

|  | β | *t* | *df*s | *p* | 95% CI |
| --- | --- | --- | --- | --- | --- |
| Eye Visibility | -0.02 | -0.76 | 2092 | .443 | [-0.08, 0.03] |
| Target Type | 0.20 | 1.00 | 302 | .314 | [-0.19, 0.61] |
| Nurturance | 0.36 | 7.61 | 301 | < .001 | [0.26, 0.45] |
| Protection | 0.30 | 6.54 | 301 | < .001 | [0.21, 0.40] |
| Interaction of Visibility and Target Type | 0.04 | 1.29 | 2092 | .195 | [-0.02, 0.10] |
| Interaction of Visibility and Nurturance | -0.02 | -1.02 | 2092 | .304 | [-0.07, 0.02] |
| Interaction of Target Type and Nurturance | -0.55 | -3.19 | 301 | .001 | [-0.90, -0.21] |
| Interaction of Visibility and Protection | 0.08 | 2.47 | 2093 | .013 | [0.01, 0.14] |
| Interaction of Target Type and Protection | 0.15 | 0.70 | 301 | .483 | [-0.27, 0.57] |
| Interaction of Visibility, Type, and Nurturance | 0.00 | 0.16 | 2092 | .868 | [-0.04, 0.05] |
| Interaction of Visibility, Type, and Protection | -0.02 | -0.63 | 2093 | .528 | [-0.08, 0.04] |
