# Supplementary material for: “Parental” responses to human infants (and puppy dogs): Evidence that the perception of eyes is especially influential, but eye contact is not
Source: PLoS One. 2020 May 6;15(5):e0232059. doi: 10.1371/journal.pone.0232059 (PMC7202593; doi:10.1371/journal.pone.0232059)
Supplement: S11 Table — (DOCX) [file pone.0232059.s011.docx]

**S11 Table. Mixed-Effects Models for Effects of Eye Visibility and Target Type on Ratings in Experiment 3.**

|  | β | *t* | *df*s | *p* | 95% CI |
| --- | --- | --- | --- | --- | --- |
| Cuteness |  |  |  |  |  |
| Eye Visibility | 0.07 | 4.68 | 833 | < .001 | [0.04, 0.11] |
| Target Type | 0.09 | 1.78 | 90 | .077 | [-0.009, 0.20] |
| Interaction | -0.13 | -8.18 | 833 | < .001 | [-0.17, -0.10] |
| Vulnerability |  |  |  |  |  |
| Eye Visibility | 0.04 | 2.83 | 833 | < .001 | [0.01, 0.07] |
| Target Type | -0.23 | -4.51 | 190 | < .001 | [-0.33, -0.13] |
| Interaction | 0.01 | 0.81 | 833 | .414 | [-0.01, 0.04] |
| Need to Protect |  |  |  |  |  |
| Eye Visibility | 0.02 | 1.97 | 833 | .048 | [0.0002, 0.05] |
| Target Type | -0.11 | -2.07 | 180 | .039 | [-0.22, -0.006] |
| Interaction | -0.01 | -0.76 | 833 | .441 | [-0.03, 0.01] |
