# Supplementary material for: “Parental” responses to human infants (and puppy dogs): Evidence that the perception of eyes is especially influential, but eye contact is not
Source: PLoS One. 2020 May 6;15(5):e0232059. doi: 10.1371/journal.pone.0232059 (PMC7202593; doi:10.1371/journal.pone.0232059)
Supplement: S12 Table — (DOCX) [file pone.0232059.s012.docx]

**S12 Table. Mixed-Effects Model for Moderating Effects of Parental Care and Tenderness on Cuteness in Experiment 3.**

|  | β | *t* | *df*s | *p* | 95% CI |
| --- | --- | --- | --- | --- | --- |
| Eye Visibility | 0.03 | 0.42 | 840 | .674 | [-0.11, 0.18] |
| Target Type | 0.53 | 2.49 | 281 | .013 | [0.11, 0.94] |
| Nurturance | 0.20 | 3.67 | 279 | < .001 | [0.09, 0.30] |
| Protection | 0.10 | 2.01 | 279 | .044 | [0.003, 0.21] |
| Interaction of Visibility and Target Type | -0.17 | -2.34 | 840 | .019 | [-0.32, -0.02] |
| Interaction of Visibility and Nurturance | -0.23 | -3.43 | 835 | < .001 | [-0.37, -0.10] |
| Interaction of Target Type and Nurturance | -0.74 | -3.82 | 279 | < .001 | [-1.13, -0.36] |
| Interaction of Visibility and Protection | 0.28 | 3.46 | 839 | < .001 | [0.12, 0.44] |
| Interaction of Target Type and Protection | 0.30 | 1.34 | 279 | .181 | [-0.14, 0.76] |
| Interaction of Visibility, Type, and Nurturance | -0.17 | -2.48 | 835 | .013 | [-0.30, -0.03] |
| Interaction of Visibility, Type, and Protection | 0.21 | 2.58 | 839 | .009 | [0.05, 0.37] |
