# Supplementary material for: “Parental” responses to human infants (and puppy dogs): Evidence that the perception of eyes is especially influential, but eye contact is not
Source: PLoS One. 2020 May 6;15(5):e0232059. doi: 10.1371/journal.pone.0232059 (PMC7202593; doi:10.1371/journal.pone.0232059)
Supplement: S13 Table — (DOCX) [file pone.0232059.s013.docx]

**S13 Table. Mixed-Effects Model for Moderating Effects of Parental Care and Tenderness on Vulnerability in Experiment 3.**

|  | β | *t* | *df*s | *p* | 95% CI |
| --- | --- | --- | --- | --- | --- |
| Eye Visibility | 0.10 | 1.44 | 835 | .150 | [-0.03, 0.24] |
| Target Type | -0.08 | -0.40 | 279 | .63 | [-0.51, 0.33] |
| Nurturance | 0.24 | 4.28 | 280 | < .001 | [0.13, 0.35] |
| Protection | 0.07 | 1.43 | 280 | .153 | [-0.02, 0.18] |
| Interaction of Visibility and Target Type | -0.09 | -1.27 | 835 | .202 | [-0.23, 0.04] |
| Interaction of Visibility and Nurturance | -0.11 | -1.74 | 838 | .080 | [-0.24, 0.01] |
| Interaction of Target Type and Nurturance | -0.20 | -1.04 | 280 | .299 | [-0.60, 0.18] |
| Interaction of Visibility and Protection | 0.05 | 0.71 | 836 | .477 | [-0.09, 0.21] |
| Interaction of Target Type and Protection | 0.07 | 0.31 | 280 | .751 | [-0.38, 0.53] |
| Interaction of Visibility, Type, and Nurturance | -0.004 | -0.06 | 838 | .950 | [-0.13, 0.12] |
| Interaction of Visibility, Type, and Protection | 0.11 | 1.42 | 836 | .156 | [-0.04, 0.26] |
