# Supplementary material for: “Parental” responses to human infants (and puppy dogs): Evidence that the perception of eyes is especially influential, but eye contact is not
Source: PLoS One. 2020 May 6;15(5):e0232059. doi: 10.1371/journal.pone.0232059 (PMC7202593; doi:10.1371/journal.pone.0232059)
Supplement: S14 Table — (DOCX) [file pone.0232059.s014.docx]

**S14 Table. Mixed-Effects Model for Moderating Effects of Parental Care and Tenderness on Need to Protect in Experiment 3.**

|  | β | *t* | *df*s | *p* | 95% CI |
| --- | --- | --- | --- | --- | --- |
| Eye Visibility | 0.11 | 1.91 | 840 | .055 | [-0.002, 0.23] |
| Target Type | -0.002 | -0.01 | 281 | .991 | [-0.43, 0.42] |
| Nurturance | 0.34 | 6.19 | 279 | < .001 | [0.23, 0.45] |
| Protection | 0.11 | 2.13 | 279 | .033 | [0.009, 0.22] |
| Interaction of Visibility and Target Type | -0.02 | -0.46 | 840 | .639 | [-0.14, 0.09] |
| Interaction of Visibility and Nurturance | -0.16 | -3.01 | 836 | .002 | [-0.27, -0.05] |
| Interaction of Target Type and Nurturance | -0.36 | -1.82 | 279 | .068 | [-0.76, 0.02] |
| Interaction of Visibility and Protection | 0.07 | 1.12 | 840 | .261 | [-0.05, 0.20] |
| Interaction of Target Type and Protection | 0.26 | 1.12 | 279 | .261 | [-0.19, 0.73] |
| Interaction of Visibility, Type, and Nurturance | -0.08 | -1.50 | 836 | .132 | [-0.19, 0.02] |
| Interaction of Visibility, Type, and Protection | 0.10 | 1.52 | 840 | .127 | [-0.02, 0.22] |
