# Supplementary material for: “Parental” responses to human infants (and puppy dogs): Evidence that the perception of eyes is especially influential, but eye contact is not
Source: PLoS One. 2020 May 6;15(5):e0232059. doi: 10.1371/journal.pone.0232059 (PMC7202593; doi:10.1371/journal.pone.0232059)
Supplement: S15 Table — (DOCX) [file pone.0232059.s015.docx]

**S15 Table. Mixed-Effects Model for Effects of Gaze Aversion and Target Type on Ratings in Experiment 4.**

|  | β | *t* | *df*s | *p* | 95% CI |
| --- | --- | --- | --- | --- | --- |
| Cuteness |  |  |  |  |  |
| Gaze Aversion | -0.13 | -2.24 | 854 | .025 | [-0.05, -0.003] |
| Target Type | 0.18 | 0.64 | 81 | .501 | [-0.07, 0.16] |
| Interaction | -0.63 | -5.34 | 854 | < .001 | [-0.09, -0.04] |
| Vulnerability |  |  |  |  |  |
| Eye Visibility | -0.009 | -0.73 | 854 | .464 | [-0.03, 0.01] |
| Target Type | -0.44 | -7.75 | 51 | < .001 | [-0.56, -0.33] |
| Interaction | 0.00 | 0.04 | 854 | .966 | [-0.02, 0.02] |
| Need to Protect |  |  |  |  |  |
| Eye Visibility | -0.02 | -2.50 | 854 | .012 | [-0.04, -0.004] |
| Target Type | -0.17 | -2.79 | 112 | .006 | [-0.29, -0.05] |
| Interaction | -0.02 | -2.86 | 854 | .004 | [-0.04, -0.008] |
