# Supplementary material for: “Parental” responses to human infants (and puppy dogs): Evidence that the perception of eyes is especially influential, but eye contact is not
Source: PLoS One. 2020 May 6;15(5):e0232059. doi: 10.1371/journal.pone.0232059 (PMC7202593; doi:10.1371/journal.pone.0232059)
Supplement: S16 Table — (DOCX) [file pone.0232059.s016.docx]

**S16 Table. Mixed-Effects Model for Moderating Effects of Parental Care and Tenderness on Cuteness in Experiment 4.**

|  | β | *t* | *df*s | *p* | 95% CI |
| --- | --- | --- | --- | --- | --- |
| Gaze Aversion | -0.02 | -0.42 | 858 | .672 | [-0.12, 0.08 |
| Target Type | 1.04 | 6.14 | 288 | < .001 | [0.71, 1.37] |
| Nurturance | 0.44 | 8.46 | 286 | < .001 | [0.34, 0.54] |
| Protection | 0.06 | 1.28 | 286 | .199 | [-0.03, 0.17] |
| Interaction of Aversion and Target Type | -0.13 | -2.42 | 858 | .015 | [-0.23, -0.02] |
| Interaction of Aversion and Nurturance | 0.07 | 1.32 | 859 | .185 | [-0.03, 0.19] |
| Interaction of Target Type and Nurturance | -0.93 | -5.17 | 286 | < .001 | [-1.29, -0.58] |
| Interaction of Aversion and Protection | -0.08 | -1.28 | 860 | .200 | [-0.20, 0.04] |
| Interaction of Target Type and Protection | -0.08 | -0.40 | 286 | .685 | [-0.47, 0.30] |
| Interaction of Aversion, Type, and Nurturance | -0.02 | -0.40 | 859 | .685 | [-0.13, 0.09] |
| Interaction of Aversion, Type, and Protection | 0.08 | 1.31 | 860 | .189 | [-0.04, 0.20] |
