# Supplementary material for: “Parental” responses to human infants (and puppy dogs): Evidence that the perception of eyes is especially influential, but eye contact is not
Source: PLoS One. 2020 May 6;15(5):e0232059. doi: 10.1371/journal.pone.0232059 (PMC7202593; doi:10.1371/journal.pone.0232059)
Supplement: S17 Table — (DOCX) [file pone.0232059.s017.docx]

**S17 Table. Mixed-Effects Model for Moderating Effects of Parental Care and Tenderness on Vulnerability in Experiment 4.**

|  | β | *t* | *df*s | *p* | 95% CI |
| --- | --- | --- | --- | --- | --- |
| Gaze Aversion | -0.06 | -1.21 | 857 | .226 | [-0.15, 0.03] |
| Target Type | -0.52 | -2.85 | 288 | .004 | [-0.87, -0.16] |
| Nurturance | 0.13 | 2.38 | 286 | .017 | [0.02, 0.24] |
| Protection | 0.16 | 2.85 | 286 | .004 | [0.05, 0.27] |
| Interaction of Aversion and Target Type | -0.03 | -0.60 | 857 | .544 | [-0.12, 0.06] |
| Interaction of Aversion and Nurturance | 0.01 | 0.22 | 858 | .825 | [-0.09, 0.11] |
| Interaction of Target Type and Nurturance | -0.38 | -1.97 | 286 | .048 | [-0.76, -0.003] |
| Interaction of Aversion and Protection | 0.04 | 0.71 | 859 | .476 | [-0.07, 0.15] |
| Interaction of Target Type and Protection | 0.48 | 2.25 | 286 | .024 | [0.06, 0.89] |
| Interaction of Aversion, Type, and Nurturance | -0.007 | -0.13 | 858 | .895 | [-0.11, 0.09] |
| Interaction of Aversion, Type, and Protection | 0.04 | 0.67 | 859 | .498 | [-0.07, 0.15] |
