# Supplementary material for: “Parental” responses to human infants (and puppy dogs): Evidence that the perception of eyes is especially influential, but eye contact is not
Source: PLoS One. 2020 May 6;15(5):e0232059. doi: 10.1371/journal.pone.0232059 (PMC7202593; doi:10.1371/journal.pone.0232059)
Supplement: S18 Table — (DOCX) [file pone.0232059.s018.docx]

**S18 Table. Mixed-Effects Model for Moderating Effects of Parental Care and Tenderness on Need to Protect in Experiment 4.**

|  | β | *t* | *df*s | *p* | 95% CI |
| --- | --- | --- | --- | --- | --- |
| Gaze Aversion | -0.03 | -1.01 | 857 | .311 | [-0.11, 0.03] |
| Target Type | 0.64 | 3.71 | 291 | < .001 | [0.30, 0.98] |
| Nurturance | 0.35 | 6.68 | 286 | < .001 | [0.25, 0.46] |
| Protection | 0.25 | 4.71 | 286 | < .001 | [0.14, 0.36] |
| Interaction of Aversion and Target Type | -0.05 | -1.45 | 857 | .146 | [-0.12, 0.01] |
| Interaction of Aversion and Nurturance | -0.03 | -0.82 | 858 | .409 | [-0.11, 0.04] |
| Interaction of Target Type and Nurturance | -0.82 | -4.45 | 286 | < .001 | [-1.18, -0.46] |
| Interaction of Aversion and Protection | 0.04 | 1.10 | 858 | .270 | [-0.03, 0.13] |
| Interaction of Target Type and Protection | 0.01 | 0.07 | 286 | .943 | [-0.38, 0.41] |
| Interaction of Aversion, Type, and Nurturance | -0.01 | -0.26 | 858 | .791 | [-0.08, 0.06] |
| Interaction of Aversion, Type, and Protection | 0.03 | 0.90 | 858 | .368 | [-0.04, 0.12] |
