# Supplementary material for: “Parental” responses to human infants (and puppy dogs): Evidence that the perception of eyes is especially influential, but eye contact is not
Source: PLoS One. 2020 May 6;15(5):e0232059. doi: 10.1371/journal.pone.0232059 (PMC7202593; doi:10.1371/journal.pone.0232059)
Supplement: S19 Table — (DOCX) [file pone.0232059.s019.docx]

**S19 Table. Mixed-Effects Model for Effects of Gaze Aversion and Target Type on Ratings in Experiment 5.**

|  | β | *t* | *df*s | *p* | 95% CI |
| --- | --- | --- | --- | --- | --- |
| Cuteness |  |  |  |  |  |
| Eye Visibility | -0.005 | -0.44 | 846 | .659 | [-0.02, 0.01] |
| Target Type | 0.24 | 4.22 | 104 | < .001 | [0.13, 0.36] |
| Interaction | 0.00 | 0.54 | 846 | .587 | [-0.01, 0.03] |
| Vulnerability |  |  |  |  |  |
| Eye Visibility | -0.008 | -0.65 | 847 | .510 | [-0.03, 0.01] |
| Target Type | -0.29 | -5.51 | 216 | < .001 | [-0.39, -0.18] |
| Interaction | 0.01 | 0.82 | 847 | .411 | [-0.01, 0.03] |
| Need to Protect |  |  |  |  |  |
| Eye Visibility | -0.002 | -0.25 | 846 | .796 | [-0.02, 0.01] |
| Target Type | -0.13 | -2.26 | 248 | .024 | [-0.24, -0.01] |
| Interaction | 0.00 | 0.47 | 846 | .635 | [-0.01, 0.02] |
