# Supplementary material for: “Parental” responses to human infants (and puppy dogs): Evidence that the perception of eyes is especially influential, but eye contact is not
Source: PLoS One. 2020 May 6;15(5):e0232059. doi: 10.1371/journal.pone.0232059 (PMC7202593; doi:10.1371/journal.pone.0232059)
Supplement: S20 Table — (DOCX) [file pone.0232059.s020.docx]

**S20 Table. Mixed-Effects Model for Moderating Effects of Parental Care and Tenderness on Cuteness in Experiment 5.**

|  | β | *t* | *df*s | *p* | 95% CI |
| --- | --- | --- | --- | --- | --- |
| Gaze Aversion | -0.07 | -1.35 | 847 | .174 | [-0.18, 0.03] |
| Target Type | 0.49 | 2.04 | 287 | .041 | [0.02, 0.97] |
| Nurturance | 0.21 | 3.38 | 283 | < .001 | [0.09, 0.34] |
| Protection | -0.03 | -0.50 | 283 | .612 | [-0.15, 0.09] |
| Interaction of Aversion and Target Type | -0.05 | -0.96 | 847 | .336 | [-0.16, 0.05] |
| Interaction of Aversion and Nurturance | -0.06 | -1.15 | 846 | .249 | [-0.17, 0.04] |
| Interaction of Target Type and Nurturance | -0.22 | -0.91 | 283 | .361 | [-0.71, 0.26] |
| Interaction of Aversion and Protection | 0.13 | 2.13 | 848 | .033 | [0.01, 0.26] |
| Interaction of Target Type and Protection | -0.05 | -0.19 | 283 | .847 | [-0.60, 0.50] |
| Interaction of Aversion, Type, and Nurturance | -0.06 | -1.11 | 846 | .266 | [-0.17, 0.04] |
| Interaction of Aversion, Type, and Protection | 0.12 | 1.93 | 848 | .053 | [-0.001, 0.25] |
