# Supplementary material for: “Parental” responses to human infants (and puppy dogs): Evidence that the perception of eyes is especially influential, but eye contact is not
Source: PLoS One. 2020 May 6;15(5):e0232059. doi: 10.1371/journal.pone.0232059 (PMC7202593; doi:10.1371/journal.pone.0232059)
Supplement: S21 Table — (DOCX) [file pone.0232059.s021.docx]

**S21 Table. Mixed-Effects Model for Moderating Effects of Parental Care and Tenderness on Vulnerability in Experiment 5.**

|  | β | *t* | *df*s | *p* | 95% CI |
| --- | --- | --- | --- | --- | --- |
| Gaze Aversion | -0.004 | -0.07 | 849 | .943 | [-0.12, 0.11] |
| Target Type | -0.08 | -0.34 | 284 | .732 | [-0.55, 0.38] |
| Nurturance | 0.02 | 0.35 | 283 | .722 | [-0.10, 0.14] |
| Protection | 0.13 | 2.07 | 283 | .038 | [0.00, 0.25] |
| Interaction of Aversion and Target Type | -0.03 | -0.61 | 849 | .541 | [-0.15, 0.08] |
| Interaction of Aversion and Nurturance | 0.04 | 0.73 | 848 | .464 | [-0.07, 0.16] |
| Interaction of Target Type and Nurturance | -0.12 | -0.51 | 283 | .607 | [-0.61, 0.35] |
| Interaction of Aversion and Protection | -0.04 | -0.70 | 851 | .480 | [-0.18, 0.08] |
| Interaction of Target Type and Protection | -0.09 | -0.34 | 283 | .733 | [-0.64, 0.45] |
| Interaction of Aversion, Type, and Nurturance | 0.00 | 0.15 | 848 | .875 | [-0.10, 0.12] |
| Interaction of Aversion, Type, and Protection | 0.03 | 0.53 | 851 | .590 | [-0.09, 0.17] |
