# Supplementary material for: “Parental” responses to human infants (and puppy dogs): Evidence that the perception of eyes is especially influential, but eye contact is not
Source: PLoS One. 2020 May 6;15(5):e0232059. doi: 10.1371/journal.pone.0232059 (PMC7202593; doi:10.1371/journal.pone.0232059)
Supplement: S22 Table — (DOCX) [file pone.0232059.s022.docx]

**S22 Table. Mixed-Effects Model for Moderating Effects of Parental Care and Tenderness on Need to Protect in Experiment 5.**

|  | β | *t* | *df*s | *p* | 95% CI |
| --- | --- | --- | --- | --- | --- |
| Gaze Aversion | -0.08 | -1.85 | 848 | .063 | -0.17, 0.00] |
| Target Type | -0.01 | -0.06 | 285 | .950 | [-0.49, 0.46] |
| Nurturance | 0.31 | 4.78 | 283 | < .001 | [0.18, 0.43] |
| Protection | 0.06 | 0.96 | 283 | .336 | [-0.06, 0.18] |
| Interaction of Aversion and Target Type | -0.04 | -0.94 | 848 | .346 | [-0.13, 0.04] |
| Interaction of Aversion and Nurturance | 0.04 | 0.99 | 847 | .321 | [-0.04, 0.13] |
| Interaction of Target Type and Nurturance | -0.43 | -1.75 | 283 | .080 | [-0.92, 0.05] |
| Interaction of Aversion and Protection | 0.03 | 0.71 | 850 | .472 | [-0.06, 0.14] |
| Interaction of Target Type and Protection | 0.27 | 0.98 | 283 | .324 | [-0.27, 0.83] |
| Interaction of Aversion, Type, and Nurturance | -0.01 | -0.26 | 847 | .794 | [-0.10, 0.07] |
| Interaction of Aversion, Type, and Protection | 0.05 | 1.11 | 850 | .265 | [-0.04, 0.16] |
