# Supplementary material for: “Parental” responses to human infants (and puppy dogs): Evidence that the perception of eyes is especially influential, but eye contact is not
Source: PLoS One. 2020 May 6;15(5):e0232059. doi: 10.1371/journal.pone.0232059 (PMC7202593; doi:10.1371/journal.pone.0232059)
Supplement: S1 Text — (DOCX) [file pone.0232059.s023.docx]

**Analyses testing for moderating effects of gender, parental status, and dog ownership**

**Experiment 1**

To test whether any of the effects of eye visibility were moderated by demographic variables, we conducted additional mixed-effects models that, in addition to the fixed effects and random effects identified in the manuscript, also included the following fixed effects: Three demographic variables of interest (gender (-0.5 = woman, 0.5 = man), parental status (-0.5 = non-parent, 0.5 = parent), and dog ownership (-0.5 = non-dog-owner; 0.5 = dog-owner)), as well as the two-way and three-way interactions between each of the three demographic variables and the target-type and eye visibility manipulations. The contrasts above were used for all the analyses in the present document.

Results revealed that only on ratings of cuteness and the need to protect was there any evidence of a statistically significant interaction between one of these demographic variables and the eye visibility manipulation (for all the output from the models, see Tables A, B, C, and D).

For ratings of cuteness, there was a 2-way interaction between the eye visibility manipulation and parental status, and a 2-way interaction between the eye visibility manipulation and dog ownership. A first set of post-hoc pairwise tests, correcting for multiple comparisons using Holm’s method, revealed that both parents and non-parents rated all targets as cuter if their eyes were visible vs. concealed (for parents, *t*(2136) = -2.51, *p* = .011; for non-parents, *t*(2136) = -7.21, *p* < .001), but that the eye visibility manipulation may have influenced non-parents’ ratings of cuteness more strongly.

A second set of post-hoc pairwise tests, correcting for multiple comparisons using Holm’s method, revealed that dog-owners and non-dog-owners both rated all targets as cuter if their eyes were visible vs. concealed (for dog-owners, *t*(2136) = -5.88, *p* < .001; for non-dog-owners, *t*(2136) = -3.27, *p* = .001), but that the eye visibility manipulation influenced dog-owners’ ratings of cuteness more strongly.

For ratings of the need to protect the target, there was a significant interaction between the eye visibility manipulation and gender. Post-hoc pairwise tests, correcting for multiple comparisons using Holm’s method, revealed that women rated targets as more in need of protection if their eyes were visible vs. concealed (*t*(2139) = -3.21, *p* = .001), but that the eye visibility manipulation did not affect men’s ratings of the need to protect a target (*t*(2143) = 0.71, *p* = .473).

**Table A. Model for Moderating Effects of Gender, Parental Status, and Dog Ownership on Cuteness in Experiment 1.**

|  | β | *t* | *df*s | *p* | 95% CI |
| --- | --- | --- | --- | --- | --- |
| Eye Visibility | 0.05 | 6.40 | 2127 | < .001 | [0.03, 0.07] |
| Target Type | 0.19 | 3.45 | 258 | < .001 | [0.08, 0.29] |
| Gender | -0.17 | -3.38 | 305 | < .001 | [-0.27, -0.07] |
| Parental Status | 0.11 | 2.26 | 305 | .024 | [0.01, 0.21] |
| Dog Ownership | 0.05 | 1.03 | 305 | .301 | [-.04, 0.14] |
| Interaction of Visibility and Target Type | -0.05 | -6.72 | 2127 | < .001 | [-0.07, -0.04] |
| Interaction of Visibility and Gender | -0.009 | -1.09 | 2132 | .272 | [-0.02, 0.00] |
| Interaction of Target Type and Gender | 0.05 | 1.03 | 305 | .299 | [-0.04, 0.15] |
| Interaction of Visibility and Parental Status | -0.01 | -2.10 | 2128 | .035 | [-0.03, -0.001] |
| Interaction of Target Type and Parental Status | -0.08 | -1.49 | 305 | .135 | [-0.18, 0.02] |
| Interaction of Visibility and Ownership | 0.01 | 2.26 | 2128 | .023 | [0.002, 0.03] |
| Interaction of Target Type and Ownership | 0.07 | 1.47 | 305 | .142 | [-0.02, 0.16] |
| Interaction of Visibility, Target Type, and Gender | 0.00 | 0.96 | 2132 | .335 | [-0.008, 0.025] |
| Interaction of Visibility, Target Type, and Parental Status | 0.00 | 0.15 | 2128 | .879 | [-0.01, 0.01] |
| Interaction of Visibility, Target Type, and Dog Ownership | -0.01 | -1.71 | 2128 | .087 | [-0.03, 0.00] |

**Table B. Model for Moderating Effects of Gender, Parental Status, and Dog Ownership on Vulnerability in Experiment 1.**

|  | β | *t* | *df*s | *p* | 95% CI |
| --- | --- | --- | --- | --- | --- |
| Eye Visibility | 0.02 | 3.01 | 2127 | .002 | [0.01, 0.04] |
| Target Type | -0.34 | -6.83 | 300 | < .001 | [-0.44, -0.24] |
| Gender | -0.12 | -2.55 | 305 | .011 | [-0.22, -0.02] |
| Parental Status | 0.07 | 1.46 | 305 | .143 | [-0.02, 0.16] |
| Dog Ownership | 0.07 | 1.65 | 305 | .099 | [-0.01, 0.16] |
| Interaction of Visibility and Target Type | 0.00 | 0.59 | 2127 | .550 | [-0.01, 0.02] |
| Interaction of Visibility and Gender | -0.004 | -0.50 | 2140 | .614 | [-0.02, 0,01] |
| Interaction of Target Type and Gender | -0.01 | -0.32 | 305 | .743 | [-0.11, 0.08] |
| Interaction of Visibility and Parental Status | -0.008 | -0.82 | 2131 | .410 | [-0.02, 0.01] |
| Interaction of Target Type and Parental Status | -0.01 | -0.35 | 305 | .723 | [-0.12, 0.08] |
| Interaction of Visibility and Ownership | -0.004 | -0.49 | 2131 | .620 | [-0.02, 0.01] |
| Interaction of Target Type and Ownership | 0.05 | 1.18 | 305 | .237 | [-0.03, 0.14] |
| Interaction of Visibility, Target Type, and Gender | 0.00 | 0.52 | 2140 | .603 | [-0.01, 0.02] |
| Interaction of Visibility, Target Type, and Parental Status | 0.00 | 0.69 | 2131 | .488 | [-0.01, 0.02] |
| Interaction of Visibility, Target Type, and Dog Ownership | 0.00 | 0.27 | 2131 | .786 | [-0.01, 0.02] |

**Table C. Model for Moderating Effects of Gender, Parental Status, and Dog Ownership on Self-Reliance in Experiment 1.**

|  | β | *t* | *df*s | *p* | 95% CI |
| --- | --- | --- | --- | --- | --- |
| Eye Visibility | 0.00 | 0.35 | 2127 | .722 | [-0.01, 0.01] |
| Target Type | 0.47 | 9.17 | 289 | < .001 | [0.37, 0.57] |
| Gender | 0.04 | 0.88 | 305 | .380 | [-0.05, 0.13] |
| Parental Status | 0.04 | 0.88 | 305 | .375 | [-0.05, 0.13] |
| Dog Ownership | -0.05 | -1.23 | 305 | .218 | [-0.14, 0.03] |
| Interaction of Visibility and Target Type | -0.01 | -1.59 | 2127 | .110 | [-0.02, 0.002] |
| Interaction of Visibility and Gender | 0.00 | 0.93 | 2133 | .350 | [-0.007, 0.02] |
| Interaction of Target Type and Gender | 0.04 | 0.88 | 305 | .377 | [-0.05, 0.13] |
| Interaction of Visibility and Parental Status | 0.00 | 0.46 | 2129 | .646 | [-0.01, 0.01] |
| Interaction of Target Type and Parental Status | 0.01 | 0.38 | 305 | .701 | [-0.08, 0.12] |
| Interaction of Visibility and Ownership | 0.00 | 0.48 | 2128 | .625 | [-0.01, 0.01] |
| Interaction of Target Type and Ownership | -0.01 | -0.27 | 305 | .784 | [-0.10, 0.07] |
| Interaction of Visibility, Target Type, and Gender | 0.00 | 0.35 | 2133 | .720 | [-0.01, 0.01] |
| Interaction of Visibility, Target Type, and Parental Status | -0.01 | -1.45 | 2129 | .146 | [-0.02, 0.004] |
| Interaction of Visibility, Target Type, and Dog Ownership | 0.01 | 1.71 | 2128 | .086 | [-0.001, 0.02] |

**Table D. Model for Moderating Effects of Gender, Parental Status, and Dog Ownership on Need to Protect in Experiment 1.**

|  | β | *t* | *df*s | *p* | 95% CI |
| --- | --- | --- | --- | --- | --- |
| Eye Visibility | 0.01 | 1.70 | 2127 | .088 | [-0.001, 0.02] |
| Target Type | -0.10 | -1.92 | 311 | .055 | [-0.21, 0.002] |
| Gender | -0.18 | -3.30 | 305 | .001 | [-0.28, -0.07] |
| Parental Status | 0.13 | 24.1 | 3-5 | .016 | [0.02, 0.23] |
| Dog Ownership | 0.12 | 2.35 | 305 | .019 | [0.02, 0.22] |
| Interaction of Visibility and Target Type | -0.008 | -1.24 | 2127 | .211 | [-0.02, 0.004] |
| Interaction of Visibility and Gender | -0.017 | -2.75 | 2137 | .005 | [-0.03, -0.005] |
| Interaction of Target Type and Gender | 0.03 | 0.65 | 305 | .515 | [-0.07, 0.14] |
| Interaction of Visibility and Parental Status | -0.007 | -1.05 | 2130 | .290 | [-0.02, 0.006] |
| Interaction of Target Type and Parental Status | -0.05 | -0.98 | 305 | .327 | [-0.17, 0.05] |
| Interaction of Visibility and Ownership | -0.001 | -0.26 | 2130 | .788 | [-0.013, 0.01] |
| Interaction of Target Type and Ownership | 0.03 | 0.63 | 305 | .524 | [-0.06, 0.13] |
| Interaction of Visibility, Target Type, and Gender | 0.01 | 1.67 | 2137 | .094 | [-0.001, 0.02] |
| Interaction of Visibility, Target Type, and Parental Status | 0.005 | 0.84 | 2130 | .396 | [-0.007, 0.01] |
| Interaction of Visibility, Target Type, and Dog Ownership | -0.003 | -0.49 | 2130 | .622 | [-0.01, 0.009] |

**Experiment 2**

To test whether any of the effects of eye visibility were moderated by demographic variables, we used the same analytic strategy employed in Experiment 1. Results revealed that only for ratings of cuteness was there a statistically significant interaction between one of these demographic variables and the eye visibility manipulation (for all the output from the models, see Tables E, F, G, and H). Specifically, there was a 3-way interaction between the eye visibility manipulation, target type, and gender. Post-hoc pairwise tests, correcting for multiple comparisons using Holm’s method, revealed that women rated puppy dogs as cuter when their eyes were visible vs. concealed (*t*(2093) = -4.86, *p* < .001); in contrast, the eye visibility manipulation did not influence women’s ratings of human infants (*t*(2095) = -1.68, *p* = .464), men’s ratings of puppy dogs (*t*(2095) = -1.72, *p* = .422), or men’s ratings of human infants (*t*(2093) = -2.23, *p* = .151).

**Table E. Model for Moderating Effects of Gender, Parental Status, and Dog Ownership on Cuteness in Experiment 2.**

|  | β | *t* | *df*s | *p* | 95% CI |
| --- | --- | --- | --- | --- | --- |
| Eye Visibility | 0.06 | 5.55 | 2085 | < .001 | [0.03, 0.08] |
| Target Type | -0.02 | -0.41 | 165 | .681 | [-0.13, 0.08] |
| Gender | -0.13 | -2.74 | 299 | .006 | [-0.23, -0.03] |
| Parental Status | 0.09 | 1.96 | 299 | .050 | [0.0003, 0.19] |
| Dog Ownership | 0.12 | 2.51 | 299 | .012 | [0.02, 0.21] |
| Interaction of Visibility and Target Type | 0.01 | 1.57 | 2085 | .116 | [-0.004, 0.03] |
| Interaction of Visibility and Gender | -0.01 | -1.69 | 2086 | .090 | [-0.04, 0.003] |
| Interaction of Target Type and Gender | -0.03 | -0.72 | 299 | .468 | [-0.13, 0.06] |
| Interaction of Visibility and Parental Status | 0.00 | 0.74 | 2088 | .458 | [-0.01, 0.03] |
| Interaction of Target Type and Parental Status | -0.14 | -2.90 | 299 | .003 | [-0.24, -0.04] |
| Interaction of Visibility and Ownership | -0.01 | -1.39 | 2091 | .164 | [-0.03, 0.006] |
| Interaction of Target Type and Ownership | -0.007 | -0.14 | 299 | .881 | [-0.10, 0.08] |
| Interaction of Visibility, Target Type, and Gender | -0.02 | -2.13 | 2086 | .032 | [-0.04, -0.002] |
| Interaction of Visibility, Target Type, and Parental Status | 0.00 | 0.22 | 2086 | .820 | [-0.01, 0.02] |
| Interaction of Visibility, Target Type, and Dog Ownership | -0.008 | -0.76 | 2091 | .445 | [-0.02, 0.01] |

**Table F. Model for Moderating Effects of Gender, Parental Status, and Dog Ownership on Vulnerability in Experiment 2.**

|  | β | *t* | *df*s | *p* | 95% CI |
| --- | --- | --- | --- | --- | --- |
| Eye Visibility | 0.06 | 6.299 | 2085 | < .001 | [0.04, 0.08] |
| Target Type | -0.54 | -11.49 | 146 | < .001 | [-0.63, -0.45] |
| Gender | -0.04 | -1.11 | 299 | .265 | [-0.13, 0.03] |
| Parental Status | 0.05 | 1.30 | 299 | .194 | [-0.02, 0.13] |
| Dog Ownership | 0.05 | 1.40 | 299 | .161 | [-0.02, 0.13] |
| Interaction of Visibility and Target Type | 0.05 | 5.16 | 2085 | < .001 | [0.03, 0.07] |
| Interaction of Visibility and Gender | 0.00 | 0.20 | 2086 | .839 | [-0.01, 0.02] |
| Interaction of Target Type and Gender | 0.04 | 1.05 | 299 | .294 | [-0.03, 0.13] |
| Interaction of Visibility and Parental Status | -0.003 | -0.36 | 2088 | .715 | [-0.02, 0.01] |
| Interaction of Target Type and Parental Status | 0.02 | 0.61 | 299 | .541 | [-0.05, 0.11] |
| Interaction of Visibility and Ownership | -0.003 | -0.32 | 2091 | .743 | [-0.02, 0.01] |
| Interaction of Target Type and Ownership | 0.08 | 2.02 | 299 | .044 | [0.00, 0.16] |
| Interaction of Visibility, Target Type, and Gender | 0.00 | 0.40 | 2086 | .682 | [-0.01, 0.02] |
| Interaction of Visibility, Target Type, and Parental Status | 0.00 | 0.14 | 2088 | .886 | [-0.01, 0.02] |
| Interaction of Visibility, Target Type, and Dog Ownership | -0.009 | -0.93 | 2091 | .350 | [-0.02, 0.01] |

**Table G. Model for Moderating Effects of Gender, Parental Status, and Dog Ownership on Self-Reliance in Experiment 2.**

|  | β | *t* | *df*s | *p* | 95% CI |
| --- | --- | --- | --- | --- | --- |
| Eye Visibility | -0.03 | -4.40 | 2085 | < .001 | [-0.04, -0.01] |
| Target Type | 0.63 | 14.43 | 244 | < .001 | [0.54, 0.71] |
| Gender | -0.03 | -0.81 | 299 | .417 | [-0.11, 0.04] |
| Parental Status | 0.00 | 0.05 | 299 | .953 | [-0.07, 0.08] |
| Dog Ownership | -0.06 | 1.59 | 299 | .110 | [-0.01, 0.14] |
| Interaction of Visibility and Target Type | -0.02 | -3.18 | 2085 | .001 | [-0.03, -0.009] |
| Interaction of Visibility and Gender | -0.009 | -1.16 | 2086 | .244 | [-0.02, 0.006] |
| Interaction of Target Type and Gender | -0.05 | -1.36 | 299 | .174 | [-0.14, 0.02] |
| Interaction of Visibility and Parental Status | 0.00 | 0.13 | 2089 | .895 | [-0.01, 0.01] |
| Interaction of Target Type and Parental Status | -0.4 | -1.00 | 299 | .315 | [-0.12, 0.04] |
| Interaction of Visibility and Ownership | -0.004 | -0.53 | 2093 | .596 | [-0.01, 0.01] |
| Interaction of Target Type and Ownership | -0.02 | -0.52 | 299 | .601 | [-0.10, 0.05] |
| Interaction of Visibility, Target Type, and Gender | -0.006 | -0.80 | 2086 | .422 | [-0.02, 0.009] |
| Interaction of Visibility, Target Type, and Parental Status | -0.009 | -1.18 | 2089 | .235 | [-0.02, 0.006] |
| Interaction of Visibility, Target Type, and Dog Ownership | 0.007 | 0.94 | 2093 | .344 | [-0.007, 0.02] |

**Table H. Model for Moderating Effects of Gender, Parental Status, and Dog Ownership on Need to Protect in Experiment 2.**

|  | β | *t* | *df*s | *p* | 95% CI |
| --- | --- | --- | --- | --- | --- |
| Eye Visibility | 0.03 | 4.52 | 2085 | < .001 | [0.01, 0.04] |
| Target Type | -0.20 | -3.86 | 304 | < .001 | [-0.31, -0.10] |
| Gender | -0.12 | -2.22 | 300 | .026 | [-0.22, -0.01] |
| Parental Status | 0.16 | 3.12 | 299 | .001 | [0.06, 0.27] |
| Dog Ownership | 0.11 | 2.17 | 300 | .030 | [0.01, 0.21] |
| Interaction of Visibility and Target Type | 0.02 | 3.86 | 2085 | < .001 | [0.01, 003] |
| Interaction of Visibility and Gender | -0.00 | -0.24 | 2087 | .805 | [-0.01, 0.01] |
| Interaction of Target Type and Gender | -0.06 | -1.12 | 300 | .263 | [-0.16, 0.04] |
| Interaction of Visibility and Parental Status | -0.00 | 1.29 | 2090 | .195 | [-0.004, 0.02] |
| Interaction of Target Type and Parental Status | -0.08 | -1.59 | 299 | .112 | [-0.19, 0.01] |
| Interaction of Visibility and Ownership | -0.007 | -1.12 | 2095 | .261 | [-0.02, 0.005] |
| Interaction of Target Type and Ownership | 0.09 | 1.84 | 300 | .065 | [-0.005, 0.19] |
| Interaction of Visibility, Target Type, and Gender | 0.00 | 0.14 | 2087 | .882 | [-0.01, 0.01] |
| Interaction of Visibility, Target Type, and Parental Status | 0.00 | 0.14 | 2090 | .886 | [-0.01, 0.01] |
| Interaction of Visibility, Target Type, and Dog Ownership | -0.005 | -0.77 | 2095 | .436 | [-0.01, 0.007] |

**Experiment 3**

To test whether any of the effects of eye visibility were moderated by demographic variables, we used the same analytic strategy employed in Experiments 1 and 2. Results revealed that there were statistically significant interactions between dog ownership and the eye visibility manipulation for all ratings (for all the output from the models, see Tables I, J, and K).

For ratings of cuteness, there was a 2-way interaction between the eye visibility manipulation and dog ownership. Post-hoc pairwise tests, correcting for multiple comparisons using Holm’s method, revealed that dog-owners rated targets as cuter when their eyes were visible vs. concealed (*t*(845) = -5.69, *p* < .001); in contrast, the eye visibility manipulation did not affect non-dog-owners’ ratings of targets’ cuteness (*t*(845) = -1.87, *p* = .061).

Similarly, for ratings of vulnerability, there was a 2-way interaction between the eye visibility manipulation and dog ownership. Post-hoc pairwise tests revealed that dog-owners rated targets as more vulnerable when their eyes were visible vs. concealed (*t*(847) = -3.21, *p* = .001); in contrast, the eye visibility manipulation did not affect non-dog-owners’ ratings of targets’ cuteness (*t*(847) = -0.47, *p* = .632).

Lastly, for ratings of the need to protect, there was again a 2-way interaction between the eye visibility manipulation and dog ownership. Here, this 2-way interaction was qualified by a 3-way interaction between eye visibility, dog ownership, and target type. Post-hoc pairwise tests revealed that dog-owners felt a stronger need to protect puppy dogs when their eyes were visible (*t*(846) = -3.11, *p* = .011); in contrast, the eye visibility manipulation did not affect dog-owners’ ratings of human infants (*t*(844) = -1.66, *p* = .384), non-dog-owners’ ratings of puppy dogs (*t*(845) = 1.79, *p* = .365), or non-dog-owners’ ratings of human infants (*t*(845) = -1.23, *p* = .584).

**Table I. Model for Moderating Effects of Gender, Parental Status, and Dog Ownership on Cuteness in Experiment 3.**

|  | β | *t* | *df*s | *p* | 95% CI |
| --- | --- | --- | --- | --- | --- |
| Eye Visibility | 0.09 | 5.42 | 833 | < .001 | [0.06, 0.12] |
| Target Type | 0.07 | 1.34 | 86 | .182 | [-0.03, 0.18] |
| Gender | 0.04 | 0.84 | 279 | .399 | [-0.05, 0.14] |
| Parental Status | 0.17 | 3.23 | 279 | .001 | [0.06, 0.28] |
| Dog Ownership | 0.06 | 1.34 | 279 | .179 | [-0.03, 0.16] |
| Interaction of Visibility and Target Type | -0.13 | -7.57 | 833 | < .001 | [-0.16, -0.09] |
| Interaction of Visibility and Gender | 0.03 | 1.74 | 839 | .082 | [[-0.003, 0.06] |
| Interaction of Target Type and Gender | -0.09 | -1.78 | 279 | .076 | [-0.20, 0.009] |
| Interaction of Visibility and Parental Status | 0.03 | 1.66 | 839 | .095 | [-0.005, 0.06] |
| Interaction of Target Type and Parental Status | -0.14 | -2.56 | 279 | .010 | [-0.25, -0.03] |
| Interaction of Visibility and Ownership | 0.05 | 2.96 | 838 | .003 | [0.01, 0.08] |
| Interaction of Target Type and Ownership | 0.01 | 0.23 | 279 | .818 | [-0.08, 0.11] |
| Interaction of Visibility, Target Type, and Gender | 0.01 | 0.89 | 839 | .372 | [-0.01, 0.05] |
| Interaction of Visibility, Target Type, and Parental Status | -0.01 | -0.99 | 839 | .318 | [-0.05, 0.01] |
| Interaction of Visibility, Target Type, and Dog Ownership | 0.02 | 1.71 | 838 | .086 | [-0.004, 0.06] |

**Table J. Model for Moderating Effects of Gender, Parental Status, and Dog Ownership on Vulnerability in Experiment 3.**

|  | β | *t* | *df*s | *p* | 95% CI |
| --- | --- | --- | --- | --- | --- |
| Eye Visibility | 0.04 | 2.67 | 834 | .007 | [0.01, 0.07] |
| Target Type | -0.21 | -3.96 | 186 | < .001 | [-0.31, -0.10] |
| Gender | 0.01 | 0.21 | 279 | .826 | [-0.09, 0.11] |
| Parental Status | 0.09 | 1.69 | 279 | .092 | [-0.01, 0.20] |
| Dog Ownership | 0.05 | 1.05 | 279 | .290 | [-0.04, 0.15] |
| Interaction of Visibility and Target Type | 0.01 | 0.98 | 834 | .323 | [-0.1, 0.04] |
| Interaction of Visibility and Gender | -0.01 | -0.88 | 838 | .377 | [-0.04, 0.01] |
| Interaction of Target Type and Gender | -0.003 | -0.06 | 279 | .946 | [-0.11, 0.10] |
| Interaction of Visibility and Parental Status | -0.01 | -0.84 | 839 | .399 | [-0.05, 0.02] |
| Interaction of Target Type and Parental Status | 0.00 | 0.00 | 279 | .994 | [-0.11, 0.11] |
| Interaction of Visibility and Ownership | 0.03 | 2.07 | 840 | .038 | [0.001, 0.06] |
| Interaction of Target Type and Ownership | 0.07 | 1.52 | 279 | .129 | [-0.02, 0.17] |
| Interaction of Visibility, Target Type, and Gender | 0.01 | 0.95 | 838 | .338 | [-0.01, 0.05] |
| Interaction of Visibility, Target Type, and Parental Status | -0.007 | -0.40 | 839 | .688 | [-0.04, 0.02] |
| Interaction of Visibility, Target Type, and Dog Ownership | 0.02 | 1.37 | 840 | .168 | [-0.009, 0.05] |

**Table K. Model for Moderating Effects of Gender, Parental Status, and Dog Ownership on Need to Protect in Experiment 3.**

|  | β | *t* | *df*s | *p* | 95% CI |
| --- | --- | --- | --- | --- | --- |
| Eye Visibility | 0.03 | 2.22 | 833 | .026 | [0.003, 0.05] |
| Target Type | -0.11 | -1.96 | 176 | .051 | [-0.22, -0.00004] |
| Gender | -0.00 | -0.03 | 279 | .968 | [-0.11, 0.11] |
| Parental Status | 0.17 | 2.99 | 279 | .002 | [0.06, 0.29] |
| Dog Ownership | 0.09 | 1.77 | 279 | .077 | [-0.01, 0.20] |
| Interaction of Visibility and Target Type | -0.00 | -0.62 | 833 | .533 | [-0.03, 0.01] |
| Interaction of Visibility and Gender | 0.01 | 1.03 | 839 | .303 | [-0.01, 0.04] |
| Interaction of Target Type and Gender | -0.05 | -0.89 | 279 | .373 | [-0.16, 0.06] |
| Interaction of Visibility and Parental Status | -0.008 | -0.56 | 839 | .573 | [-0.03, 0.02] |
| Interaction of Target Type and Parental Status | -0.04 | -0.70 | 279 | .480 | [-0.15, 0.07] |
| Interaction of Visibility and Ownership | 0.03 | 2.81 | 839 | .005 | [0.01, 0.06] |
| Interaction of Target Type and Ownership | -0.002 | -0.05 | 279 | .960 | [-0.10, 0.10] |
| Interaction of Visibility, Target Type, and Gender | 0.00 | 0.17 | 839 | .857 | [-0.02, 0.03] |
| Interaction of Visibility, Target Type, and Parental Status | -0.01 | -1.01 | 839 | .312 | [-0.04, 0.01] |
| Interaction of Visibility, Target Type, and Dog Ownership | 0.03 | 2.31 | 839 | .020 | [0.004, 0.05] |

**Experiment 4**

To test whether any of the effects of eye gaze were moderated by demographic variables, we conducted additional mixed-effects models that, in addition to the fixed effects and random effects identified in the manuscript, also included the following fixed effects: Three demographic variables of interest (gender, parental status, and dog ownership), as well as the two-way and three-way interactions between each of the three demographic variables and the target type and eye gaze manipulations.

Results revealed that only for ratings of cuteness was there a statistically significant interaction between one of these demographic variables and the eye gaze manipulation (for all the output from the models, see Tables L, M, and N). Specifically, there was a 3-way interaction between gaze aversion, target type, and dog ownership. Post-hoc pairwise tests, correcting for multiple comparisons using Holm’s method, revealed that non-dog-owners rated puppy dogs as being cuter when gaze was direct vs. averted (*t*(864) = 4.34, *p* < .001) and human infants as being *less* cute when gaze was direct vs. averted (*t*(863) = -2.68, *p* = .029); in contrast; the gaze manipulation did not affect dog-owners’ ratings of puppy dogs (*t*(863) = 2.32, *p* = .121), or dog-owners’ ratings of human infants (*t*(866) = 0.17, *p* = 1.00).

**Table L. Model for Moderating Effects of Gender, Parental Status, and Dog Ownership on Cuteness in Experiment 4.**

|  | β | *t* | *df*s | *p* | 95% CI |
| --- | --- | --- | --- | --- | --- |
| Gaze Aversion | -0.02 | -2.06 | 855 | .039 | [-0.05, -0.001] |
| Target Type | -0.03 | -0.50 | 75 | .612 | [-0.14, 0.08] |
| Gender | -0.19 | -3.92 | 286 | < .001 | [-0.29, -0.09] |
| Parental Status | 0.05 | 1.15 | 286 | .247 | [-0.04, 0.16] |
| Dog Ownership | 0.01 | 0.38 | 286 | .700 | [-0.07, 0.11] |
| Interaction of Aversion and Target Type | -0.06 | -4.42 | 855 | < .001 | [-0.09, -0.03] |
| Interaction of Aversion and Gender | -0.01 | -1.16 | 857 | .243 | [-0.04, 0.01] |
| Interaction of Target Type and Gender | 0.03 | 0.69 | 286 | .488 | [-0.06, 0.13] |
| Interaction of Aversion and Parental Status | -0.006 | -0.41 | 857 | .678 | [-0.03, 0.02] |
| Interaction of Target Type and Parental Status | -0.29 | -5.44 | 286 | < .001 | [-0.39, -0.18] |
| Interaction of Aversion and Ownership | -0.006 | -0.51 | 856 | .608 | [-0.03, 0.01] |
| Interaction of Target Type and Ownership | 0.13 | 2.74 | 286 | .006 | [0.03, 0.23] |
| Interaction of Aversion, Target Type, and Gender | -0.008 | -0.60 | 857 | .543 | [-0.03, 0.01] |
| Interaction of Aversion, Target Type, and Parental Status | 0.00 | 0.41 | 857 | .675 | [-0.02, 0.03] |
| Interaction of Aversion, Target Type, and Dog Ownership | 0.03 | 2.32 | 856 | .020 | [0.004, 0.05] |

**Table M. Model for Moderating Effects of Gender, Parental Status, and Dog Ownership on Vulnerability in Experiment 4.**

|  | β | *t* | *df*s | *p* | 95% CI |
| --- | --- | --- | --- | --- | --- |
| Gaze Aversion | -0.009 | -0.73 | 855 | .463 | [-0.03, 0.01] |
| Target Type | -0.48 | -8.34 | 56 | < .001 | [-0.60, -0.37] |
| Gender | -0.13 | -2.79 | 286 | .005 | [-0.22, -0.03] |
| Parental Status | 0.07 | 1.56 | 286 | .117 | [-0.01, 0.17] |
| Dog Ownership | 0.008 | 0.17 | 286 | .862 | [-0.08, 0.09] |
| Interaction of Aversion and Target Type | 0.002 | 0.19 | 855 | .842 | [-0.02, 0.02] |
| Interaction of Aversion and Gender | -0.01 | -1.22 | 856 | .219 | [-0.04, 0.009] |
| Interaction of Target Type and Gender | 0.07 | 1.55 | 286 | .121 | [-0.01, 0.17] |
| Interaction of Aversion and Parental Status | -0.008 | -0.65 | 857 | .516 | [-0.03, 0.01] |
| Interaction of Target Type and Parental Status | -0.09 | -1.92 | 286 | .055 | [-0.19, 0.001] |
| Interaction of Aversion and Ownership | -0.003 | -0.25 | 855 | .798 | [-0.02, 0.02] |
| Interaction of Target Type and Ownership | -0.006 | -0.13 | 286 | .896 | [-0.09, 0.08] |
| Interaction of Aversion, Target Type, and Gender | 0.00 | 0.36 | 856 | .712 | [-0.02, 0.03] |
| Interaction of Aversion, Target Type, and Parental Status | 0.00 | 0.41 | 857 | .75 | [-0.02, 0.03] |
| Interaction of Aversion, Target Type, and Dog Ownership | -0.002 | -0.16 | 855 | .865 | [-0.02, 0.02] |

**Table N. Model for Moderating Effects of Gender, Parental Status, and Dog Ownership on Need to Protect in Experiment 4.**

|  | β | *t* | *df*s | *p* | 95% CI |
| --- | --- | --- | --- | --- | --- |
| Gaze Aversion | -0.02 | -2.28 | 855 | .022 | [-0.04, -0.003] |
| Target Type | -0.25 | -4.34 | 107 | < .001 | [-0.37, -0.14] |
| Gender | -0.22 | -4.42 | 286 | < .001 | [-0.33, -0.12] |
| Parental Status | 0.08 | 1.69 | 286 | .091 | [-0.01, 0.19] |
| Dog Ownership | 0.06 | 1.37 | 286 | .170 | [-0.02, 0.16] |
| Interaction of Aversion and Target Type | -0.02 | -2.52 | 855 | .011 | [-0.04, -0.005] |
| Interaction of Aversion and Gender | -0.01 | -1.79 | 856 | .073 | [-0.03, 0.001] |
| Interaction of Target Type and Gender | 0.04 | 0.85 | 286 | .393 | [-0.05, 0.14] |
| Interaction of Aversion and Parental Status | -0.007 | -0.77 | 856 | .438 | [-0.02, 0.01] |
| Interaction of Target Type and Parental Status | -0.29 | -5.45 | 286 | < .001 | [-0.40, -0.19] |
| Interaction of Aversion and Ownership | -0.004 | -0.49 | 855 | .617 | [-0.02, 0.01] |
| Interaction of Target Type and Ownership | 0.04 | 0.89 | 286 | .371 | [-0.05, 0.14] |
| Interaction of Aversion, Target Type, and Gender | -0.001 | -0.13 | 856 | .896 | [-0.02, 0.01] |
| Interaction of Aversion, Target Type, and Parental Status | -0.002 | -0.21 | 856 | .827 | [-0.02, 0.01] |
| Interaction of Aversion, Target Type, and Dog Ownership | 0.00 | 0.44 | 855 | .654 | [-0.01, 0.02] |

**Experiment 5**

To test whether any of the effects of eye gaze were moderated by demographic variables, we used the same analytic strategy employed in Experiment 4. Results revealed that that there was a statistically significant interaction between dog ownership and the eye gaze manipulation for ratings of vulnerability and the need to protect (for all the output from the models, see Tables O, P, and Q).

For ratings of vulnerability, there was a 2-way interaction between gaze aversion and dog ownership. Post-hoc pairwise tests, correcting for multiple comparisons using Holm’s method, revealed that non-dog-owners rated targets as being more vulnerable when gaze was direct vs. averted (*t*(849) = 2.54, *p* = .011); in contrast, there was no effect of the gaze manipulation on dog-owners’ ratings (*t*(847) = -1.66, *p* = .097).

For ratings of the need to protect, there was a 2-way interaction between gaze aversion and dog ownership. After correcting for multiple comparisons using Holm’s method, however, there was no effect of the gaze manipulation on dog-owners’ ratings (*t*(847) = -1.13, *p* = .258), or on non-dog-owners’ ratings (*t*(848) = 1.71, *p* = .086).

**Table O. Model for Moderating Effects of Gender, Parental Status, and Dog Ownership on Cuteness in Experiment 5.**

|  | β | *t* | *df*s | *p* | 95% CI |
| --- | --- | --- | --- | --- | --- |
| Gaze Aversion | -0.007 | -0.61 | 837 | .539 | [-0.03, 0.01] |
| Target Type | 0.210 | 3.55 | 101 | < .001 | [0.09, 0.32] |
| Gender | 0.005 | 0.09 | 280 | .921 | [-0.10, 0.11] |
| Parental Status | 0.095 | 1.73 | 280 | .084 | [-0.01, 0.20] |
| Dog Ownership | 0.160 | 2.90 | 280 | .003 | [0.05, 0.26] |
| Interaction of Aversion and Target Type | 0.005 | 0.46 | 837 | .640 | [-0.01, 0.03] |
| Interaction of Aversion and Gender | -0.0002 | -0.02 | 839 | .981 | [-0.02, 0.02] |
| Interaction of Target Type and Gender | -0.04 | -0.87 | 280 | .383 | [-0.15, 0.06] |
| Interaction of Aversion and Parental Status | 0.006 | 0.49 | 839 | .617 | [-0.01, 0.03] |
| Interaction of Target Type and Parental Status | -0.08 | -1.63 | 280 | .103 | [-0.19, 0.01] |
| Interaction of Aversion and Ownership | 0.01 | 1.08 | 839 | .277 | [-0.01, 0.03] |
| Interaction of Target Type and Ownership | 0.00 | 0.13 | 280 | .896 | [-0.10, 0.11] |
| Interaction of Aversion, Target Type, and Gender | -0.0003 | -0.02 | 839 | .979 | [-0.02, 0.02] |
| Interaction of Aversion, Target Type, and Parental Status | 0.00 | 0.32 | 839 | .742 | [-0.02, 0.02] |
| Interaction of Aversion, Target Type, and Dog Ownership | 0.02 | 1.86 | 839 | .062 | [-0.00, 0.04] |

**Table P. Model for Moderating Effects of Gender, Parental Status, and Dog Ownership on Vulnerability in Experiment 5.**

|  | β | *t* | *df*s | *p* | 95% CI |
| --- | --- | --- | --- | --- | --- |
| Gaze Aversion | -0.007 | -0.53 | 837 | .591 | [-0.03, 0.01] |
| Target Type | -0.31 | 5.82 | 210 | < .001 | [-0.42, -0.20] |
| Gender | 0.01 | 0.21 | 280 | .829 | [-0.09, 0.11] |
| Parental Status | 0.07 | 1.45 | 280 | .147 | [-0.02, 0.18] |
| Dog Ownership | 0.08 | 1.50 | 280 | .132 | [-0.02, 0.19] |
| Interaction of Aversion and Target Type | 0.008 | 0.65 | 837 | .512 | [-0.01, 0.03] |
| Interaction of Aversion and Gender | 0.008 | 0.64 | 842 | .519 | [-0.13, 0.07] |
| Interaction of Target Type and Gender | -0.03 | -0.57 | 280 | .568 | [-0.13, 0.07] |
| Interaction of Aversion and Parental Status | -0.01 | -0.83 | 842 | .401 | [-0.03, 0.01] |
| Interaction of Target Type and Parental Status | -0.04 | -0.75 | 280 | 4.51 | [-0.14, 0.06] |
| Interaction of Aversion and Ownership | 0.03 | 2.91 | 842 | .003 | [0.01, 0.06] |
| Interaction of Target Type and Ownership | 0.01 | 0.35 | 280 | .719 | [-0.08, 0.12] |
| Interaction of Aversion, Target Type, and Gender | -0005 | -0.37 | 842 | .711 | [-0.03, 0.02] |
| Interaction of Aversion, Target Type, and Parental Status | 0.007 | 0.56 | 842 | .570 | [-0.01, 0.03] |
| Interaction of Aversion, Target Type, and Dog Ownership | 0.001 | 0.09 | 842 | .926 | [-0.02, 0.02] |

**Table Q. Model for Moderating Effects of Gender, Parental Status, and Dog Ownership on Need to Protect in Experiment 5.**

|  | β | *t* | *df*s | *p* | 95% CI |
| --- | --- | --- | --- | --- | --- |
| Gaze Aversion | -0.003 | -0.35 | 837 | .723 | [-0.02, 0.01] |
| Target Type | -0.16 | -2.90 | 241 | .004 | [-0.27, -0.05] |
| Gender | -0.08 | -1.53 | 280 | .126 | [-0.19, 0.02] |
| Parental Status | 0.15 | 2.78 | 280 | .005 | [0.04, 0.27] |
| Dog Ownership | 0.10 | 1.90 | 280 | .058 | [-0.003, 0.22] |
| Interaction of Aversion and Target Type | 0.001 | 0.17 | 837 | .857 | [-0.01, 0.02] |
| Interaction of Aversion and Gender | -0.007 | -0.69 | 841 | .487 | [-0.02, 0.01] |
| Interaction of Target Type and Gender | -0.02 | -0.42 | 280 | .668 | [-0.13, 0.08] |
| Interaction of Aversion and Parental Status | -0.007 | -0.73 | 841 | .464 | [-0.02, 0.01] |
| Interaction of Target Type and Parental Status | -0.10 | -1.85 | 280 | .064 | [-0.21, 0.005] |
| Interaction of Aversion and Ownership | 0.02 | 1.97 | 841 | .048 | [0.0001, 0.04] |
| Interaction of Target Type and Ownership | 0.10 | 1.90 | 280 | .057 | [-0.003, 0.22] |
| Interaction of Aversion, Target Type, and Gender | -0.008 | -0.78 | 841 | .433 | [-0.02, 0.01] |
| Interaction of Aversion, Target Type, and Parental Status | 0.00 | 0.03 | 841 | .974 | [-0.01, 0.02] |
| Interaction of Aversion, Target Type, and Dog Ownership | 0.00 | 0.96 | 841 | .335 | [-0.01, 0.03] |
